# The core-promoter factor TRF2 mediates a Fruitless action to masculinize neurobehavioral traits in *Drosophila*

**DOI:** 10.1038/s41467-017-01623-z

**Published:** 2017-11-14

**Authors:** Zahid Sadek Chowdhury, Kosei Sato, Daisuke Yamamoto

**Affiliations:** Tohoku University Graduate School of Life Sciences, Sendai, 9808577 Japan

## Abstract

In fruit flies, the male-specific *fruitless* (*fru*) gene product FruBM plays a central role in establishing the neural circuitry for male courtship behavior by orchestrating the transcription of genes required for the male-type specification of individual neurons. We herein identify the core promoter recognition factor gene *Trf2* as a dominant modifier of *fru* actions. *Trf2* knockdown in the sexually dimorphic mAL neurons leads to the loss of a male-specific neurite and a reduction in male courtship vigor. TRF2 forms a repressor complex with FruBM, strongly enhancing the repressor activity of FruBM at the promoter region of the *robo1* gene, whose function is required for inhibiting the male-specific neurite formation. In females that lack FruBM, TRF2 stimulates *robo1* transcription. Our results suggest that TRF2 switches its own role from an activator to a repressor of transcription upon binding to FruBM, thereby enabling the ipsilateral neurite formation only in males.

## Abstract (web summary)

*fruitless* (*fru*) is an important sex-determinant gene that controls the expression of neuroanatomical sex differences in *Drosophila*. Here the authors report that a core-promoter factor, TRF2, suppresses a male-type neurite specification through direct interaction with FruBM isoform at the *robo1* target gene promoter.

## Introduction

The female and male of a sexually reproducing animal are, in principle, different from each other in structure and function at the molecular, cellular, and organismal levels. Sexual traits often represent the most striking variations within a species, as a result of strong pressure from sexual selection^1^. The fruit fly *Drosophila melanogaster*, an excellent model for studying the genetic organization of complex traits, shows remarkable sexual dimorphisms in many aspects of its structure and function, including its behavior^2^. To court females, males of *D. melanogaster* engage in a series of sophisticated behavioral actions, which include orientation toward the target, chasing, tapping, wing extension/vibration for love song generation, licking and attempted copulation, culminating in copulation^3–6^. Normal females do not show any of these male-typical behaviors and, instead, signify rejection of male courtship by decamping, fending, flickering the wings, kicking, curling the abdomen and extruding the ovipositor, or signify their acceptance by slowing down locomotion, raising their wings, and opening the vaginal plate^7–11^. These sexual differences in mating behavior reflect sexual differences in the nervous system, which activates a sex-specific motor program under the circumstances where the repertoire for mating behavior rather than that for other behaviors (e.g., aggression, feeding, and sleeping) is appropriate^12^. A significant portion of the neural circuitry for mating behavior is composed of sexually dimorphic or sex-specific neurons in this species^13^. The neural sex differences in *Drosophila* are generated under the hierarchical control of two sex-determinant genes, *fruitless* (*fru*) and *doublesex* (*dsx*)^14,15^. *dsx* plays a key role for sex determination in both neural and non-neural cells, whereas *fru* exerts its sex-determinant function only in neural cells^14,15^. This neuron-specific sex-determinant role of *fru* provides us with an outstanding opportunity to unravel the causal relationship between the single neuron sex difference and the behavioral sex difference with minimal possible disturbances in the non-neural sex determination process.

The sexual function of *fru* is attributable exclusively to the P1 promoter-derived transcripts^16–18^, which produce five isoforms of Fru in males but no protein in females^19,20^. Thus the P1 promoter-derived transcripts encode male-specific Fru isoforms, FruAM, FruBM, FruCM, FruDM, and FruEM, where M stands for male-specific and A-E indicates the C-terminal variant type (isoforms A, B, and E by our nomenclature correspond to isoforms A, C, and B by the nomenclature adopted by von Philipsborn et al.^21^, respectively), among which FruBM (FruCM in von Philipsborn et al.^21^) is the isoform with the strongest impact on neurobehavioral masculinization^20–23^. FruBM has an N-terminal BTB domain and two zinc-finger motifs at the C-terminus, suggestive of its role as a transcriptional factor^17,24^. Indeed, FruBM forms a complex with proteins known to function as chromatin factors, i.e., heterochromatin protein 1a (HP1a), histone deacetylase 1 (HDAC1), and the TIF1 homolog Bonus (Bon)^25^. A large number of potential transcriptional targets of FruBM have been proposed based on DamID, ChIP, and transcriptome analyses^23,26^. However, there exists only one established target gene of FruBM, in the sense that it has a defined *cis* element for FruBM binding, and the in vivo outcome of FruBM-binding to the *cis* element has been firmly demonstrated^27^. This target gene, *robo1*, encodes a key effector that specifies the neurite sex-type of a group of *fru*-expressing interneurons called the mAL cluster^27^. The mAL cluster is sexually dimorphic in three respects^28^: the number of cells composing the cluster is 5 in females vs. 30 in males; the contralateral neurite bifurcates in females but not in males; and the ipsilateral neurite exists only in males. Loss of *fru* in males transforms the mAL cluster from the male-type into the female-type in all three respects^28^. *robo1* has been shown to inhibit the formation of the ipsilateral neurite in females, whereas, in males, male-specific FruBM represses *robo1* transcription, thereby allowing the ipsilateral neurite to form^27^. In contrast, *Hunchback* (*Hb*) is required for specification of the branching pattern of the contralateral neurite with no role in the ipsilateral neurite formation^29^. The sex difference in the mAL cell number is a result of female-specific cell death in which neither *robo1* nor *Hb* plays a role^28^. Thus, FruBM appears to regulate a distinct set of genes for establishing the sex-type of each of three sexually dimorphic structures of the mAL cluster. However, the mechanism whereby FruBM regulates a unique set of genes for a particular sexual trait while controlling another set of genes for the other sexual trait remains an enigma. In the present study, by searching for genetic modifiers of a *fru*-induced phenotype, we recover *Trf2*, a gene encoding a core promoter factor, and find that it suppresses the male-specific mAL ipsilateral neurite formation in females. We further demonstrate that TRF2 contributes to a FruBM-containing complex and regulates *robo1* transcription. Surprisingly, we find that TRF2 strongly enhances the transcription repressor activity of FruBM when it is recruited to the FruBM-containing protein complex, whereas TRF2 on its own activates *robo1* transcription in the absence of FruBM. We propose that TRF2 governs sex-specific function of a FruBM target gene, *robo1*, from that of an activator to a repressor upon detection of the presence of FruBM in males.

### Isolation of *Trf2* as a phenotypic modifier of *fru*

**Fig. 1:**
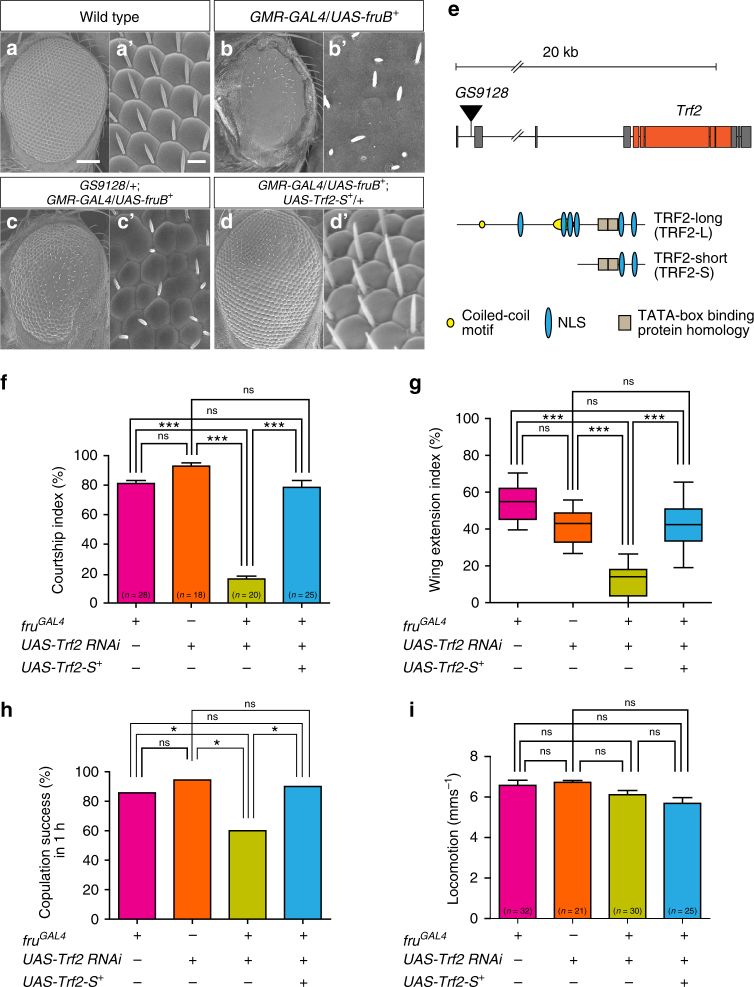
Phenotypic interactions of *Trf2* and *fru*. **a**–**d** A regular array of ommatidia in the compound eye (wild type: **a**) was distorted by *fruB* ^*+*^ overexpression (**b**), and the *fruB* ^*+*^-induced eye phenotype was partially rescued by *GS9128* (**c**) or *Trf2-S* ^*+*^ overexpression (**d**). Enlarged views are shown in **a**′–**d**′. **e** Schematic drawings of the genomic organization of the *Trf2* locus with the *GS9128* P-insertion (upper panel) and TRF2-L and TRF2-S isoforms (lower panel). **f**–**i** *Trf2-S* knockdown in *fru* ^*GAL4*^-expressing cells reduced the courtship index (**f**), wing extension index (**g**), and copulation success (**h**), but had no effect on locomotor activity (**i**) in male flies, and the effects of *Trf2* knockdown were compensated by *Trf2-S* ^*+*^ overexpression (**f**–**h**). Statistical differences were evaluated by one-way ANOVA followed by Tukey’s multiple comparison test (**f**, **i**), by the Kruskal–Wallis test followed by the Dunn’s multiple comparison test (**g**), or by the *χ* ^2^-test (**h**). ***P < 0.001, *P < 0.05; ns not significant. The number of flies examined is indicated near the base of each bar. Data shown in **f**–**h** were obtained from the same fly group, whereas data shown in **i** were derived from a fly group different from that used in **f**–**h**. Error bars indicate SEM. Scale bars: 200 µm (**a**), 30 µm (**a**′)

In screening a large collection of GS *P*-element insertion lines^30^ for possible phenotypic interactions with *fru*, we took advantage of the visible phenotype induced upon overexpression of *fru* ^*+*^ in the eye-antennal disc, which distorted the adult compound eye structure (Fig. 1a, b). In this screen, we chose to express FruB, one of the non-sex-specific Fru proteins. Although FruBM is the most prevalent isoform in neural masculinization^31^, we found that FruBM overexpression via *GMR-GAL4* led to lethality. To circumvent this problem, we used FruB. Non-sex-specific Fru proteins that are not endogenously expressed in postembryonic neurons have been shown to masculinize neurons when they are artificially expressed^20,32^. The GS system allows one to overexpress a gene flanking the GS *P*-element insertion in the presence of a *GAL4* driver^30^. In our screen, *GMR-GAL4* acted to drive both *UAS-fruB* and the second target in the genome, which flanked a GS *P*-element. After examining 1364 GS lines, we recovered 40 dominant suppressors of the eye distortion phenotype induced by *fruB* overexpression, including *GS9128* (Fig. 1c). The GS *P*-element in *GS9128* had impinged immediately 5′ to the *Trf2* transcription unit (Fig. 1e)^33^, suggesting that it was *Trf2* that was involved in the observed suppression of the *fru*-induced eye distortion. The *Trf2* gene produces the TRF2-long (TRF2-L) and TRF2-short (TRF2-S) protein isoforms^34^. The effect of *GS9128* in suppressing the *fruB*-induced distorted eye phenotype was recapitulated by overexpressing TRF2-S in the absence of *GS9128* (Fig. 1d), indicating that *Trf2* was indeed responsible for suppression of the *fru*-induced eye phenotype.

### *Trf2* supports sex-specific neurobehavioral traits

To determine whether *Trf2* plays a role in generating sex-specific traits as *fru* does, we measured male courtship behavior in flies in which the *Trf2* activity was genetically reduced by the action of *Trf2 RNAi*. We ascertained that TRF2 immunoreactivity vanishes upon the expression of *Trf2 RNAi* in cells that otherwise accumulate a large amount of TRF2 (Supplementary Fig. 1). *Trf2* knockdown in *fru* ^*GAL4*^-expressing neurons resulted in a marked reduction in the courtship index (CI) (Fig. 1f), wing extension index (WEI) (Fig. 1g), and copulation success rate (Fig. 1h), without affecting locomotion (Fig. 1i). All of the courtship defects induced by *Trf2* knockdown were completely rescued by the overexpression of a transgene encoding TRF2-S (*Trf2-S* ^*+*^; Fig. 1f–h). Female flies with *Trf2* knockdown in *fru* ^*GAL4*^-expressing neurons exhibited reduced mating success and deposited fewer eggs (Supplementary Fig. 2), indicating that *Trf2* plays a role in these female-specific functions.

**Fig. 2:**
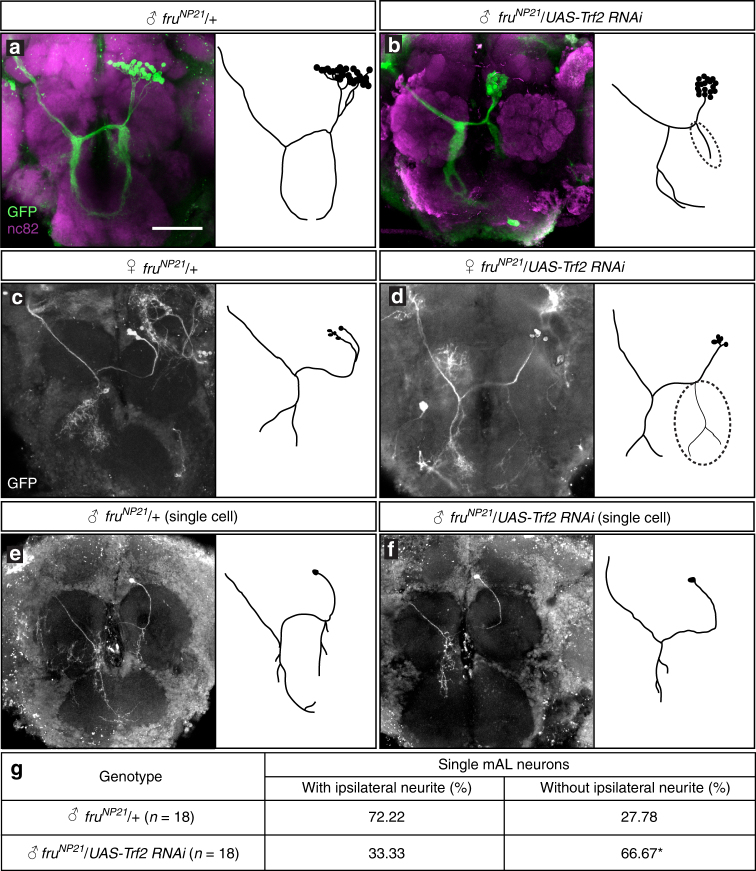
*Trf2* is required for the male-specific neurite formation in mAL neurons. **a**–**d** mAL neuroblast clones without (**a**, **c**), or with (**b**, **d**) expression of *Trf2* RNAi in male (**a**, **b**), or female (**c**, **d**) flies. **e**, **f** Single-cell clones with (**e**) or without (**f**) the ipsilateral neurite in a control male (**e**) and a male with *Trf2* knockdown (**f**). **g** Quantitative comparisons of the frequency of obtaining single-cell clones with and without the ipsilateral neurite between wild-type and *Trf2* knockdown mAL neurons in males. Statistical differences were evaluated by the *χ* ^2^-test (**g**). *P < 0.05. Scale bar: 50 µm (**a**)

Sexually dimorphic circuitries underlie gendered behavior^13^. We have therefore examined the possible effects of *Trf2* knockdown on sexually dimorphic neural structures. Here we focus on a particular neural cluster, mAL, which exhibits conspicuous sex differences in three respects, all of which depend on the presence (for masculinization) or absence (for feminization) of FruM: the number of neurons composing the cluster is 5 in females compared with 30 in males; none of the mAL neurons in females has the ipsilateral neurite, whereas some of the mAL neurons in males have it; and the contralateral neurite in the suboesophageal ganglion bifurcates near the tip in females but not in males^28^. We confirmed that all mAL neurons express TRF2 as well as FruM (Supplementary Fig. 3). To visualize the entire structure of mAL neurons, we employed the mosaic analysis with a repressible cell marker (MARCM) technique^35^, which allows one to label the mAL cluster only in the left or right hemisphere without any interference from the mAL counterpart on the other side. When the mAL cluster was visualized as a neuroblast clone, the male-specific ipsilateral neurite appeared shorter in males with *Trf2* knockdown than in control males (Fig. 2a, b). mAL neuroblast clones in *fru* hypomorphic mutant males exhibited a similar shortening of the ipsilateral neurite, which was ascribed to a reduction in the number of mAL neurons with a long ipsilateral neurite as resulting from sexual transformation of single cells^25^. To determine whether *Trf2* knockdown similarly increases the mAL neurons without the male-specific neurite at the expense of those with the male-specific neurite, we generated single-cell clones of mAL neurons. Notably, *Trf2* knockdown in mAL neurons of male flies resulted in a marked increase in the proportion of mAL neurons without the male-specific neurite, compared with control flies: 66.67% in *Trf2* knockdown vs. 33.33% in control flies (Fig. 2e–g). This effect of *Trf2* knockdown is reminiscent of the effect of a reduction in functional of FruM doses by the *fru* hypomorphic mutation (e.g., the proportion of single clones without the male-specific neurite in *fru* ^*NP21*^ */fru* ^*2*^ was 50% in ref. ^25^). We conclude that *Trf2* knockdown in mAL neurons phenocopies the *fru* mutant effect in that the mAL neurons without the male-specific neurite are produced at the expense of those with the male-specific neurite.

In contrast to the robust effect of *Trf2* knockdown on male-specific neurite formation, the shape of the contralateral neurite was marginally affected by *Trf2* knockdown. In females, *Trf2* knockdown resulted in formation of the male-specific ipsilateral neurites in three out of seven brains (Fig. 2c, d), implying that *Trf2* exerts a feminizing effect on mAL neurons in wild-type females that lack FruBM.

**Fig. 3:**
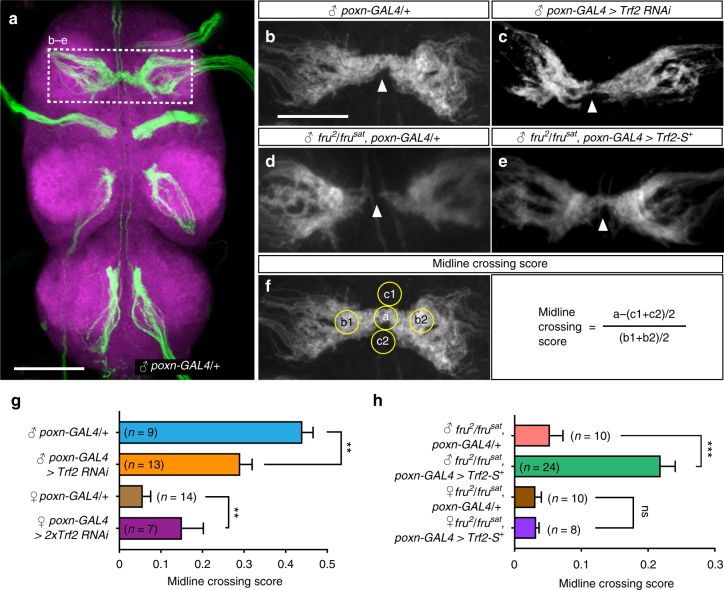
*Trf2* is required for the establishment of sexually dimorphic sensory innervation. **a**–**e** Sex-specific sensory projections in the prothoracic ganglion. **a** The sensory projections stained for *poxn-GAL4*-positive neurons in the entire ventral nerve cord of a male. Mellert et al.^36^ reported that 96% of *poxn*-*GAL4*-positive sensory cells in forelegs are *fru*-positive. The prothoracic segment is boxed with a broken line. **b**–**e** Enlarged views of prothoracic sensory projections in flies of the indicated genotype. Male-specific midline crossing of sensory afferents (wild-type male: **b**) was inhibited by *Trf2* knockdown in *poxn-GAL4*-expressing cells in a male fly (**c**). The reduced level of midline crossing of sensory fibers in the *fru* hypomorphic mutant male (**d**) was partially rescued by the overexpression of a wild-type *Trf2-S* transgene (*Trf2-S* ^*+*^) (**e**). **f** The regions examined when quantifying the fluorescent intensity to estimate the level of midline crossing of sensory fibers. For more details, see the main text and Methods. **g**, **h** Quantitative comparisons of the midline crossing score between flies with and without *Trf2* knockdown (**g**) and those with and without *Trf2-S* ^*+*^ overexpression (**h**) in males and females. Statistical differences were evaluated by Student’s *t* test. ***P < 0.001, **P < 0.01; ns not significant. Error bars indicate SEM. Scale bars: 100 µm (**a**), 50 µm (**b**)

To determine whether *Trf2* is involved in the development of *fru*-dependent sex-specific characteristics of other neurons, we labeled foreleg sensory afferents, the central projections of which cross the midline in males but not females^36,37^. The extent to which the sensory fibers cross the midline was quantified by the midline crossing score (MCS)^36^, which was based on the fluorescent intensity measured at the midline relative to that at “blank” regions where no sensory fibers run (Fig. 3f). This analysis revealed that the MCS was significantly decreased in males by *Trf2* knockdown as directed by *poxn-GAL4*, a driver for gustatory receptor neurons (Fig. 3a–c, g). Conversely, *Trf2* knockdown in females increased the midline crossing (Fig. 3g), suggesting the feminizing ability of *Trf2* in the absence of FruBM. In hypomorphic *fru* (*fru* ^*2*^ */fru* ^*sat*^) mutant males where the midline crossing was reduced (Fig. 3d, e, g), *Trf2-S* ^*+*^ overexpression partially restored the midline crossing (Fig. 3h). We conclude that *Trf2* participates in establishing neural sex-specific characteristics in two opposing ways, i.e., masculinizing neurons in the presence of FruBM and feminizing neurons in the absence of FruBM.

### TRF2-S enhances Fru-induced repression of *robo1*

**Fig. 4:**
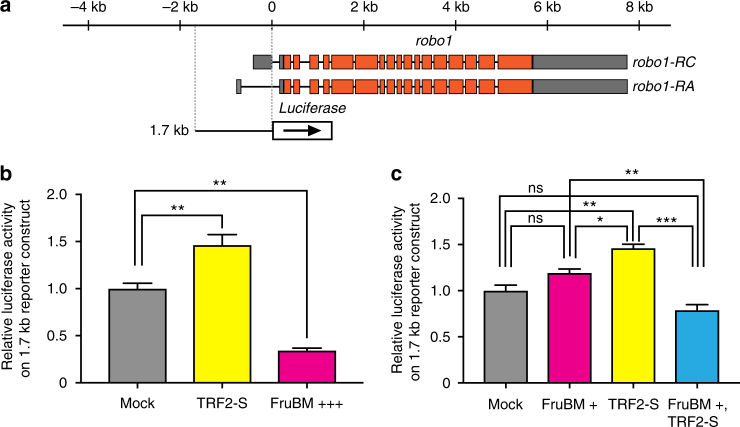
TRF2-S and FruBM regulate transcription from the *robo1* promoter. **a** Schematic representation of the exon–intron organization for two *robo1* transcripts (*robo1-RA* and *robo1-RC*) and the *robo1* reporter construct. **b** A luciferase reporter fused to the *robo1* promoter fragment of 1.7 kb exhibited an elevated transcription in the presence of TRF2-S alone and a reduced transcription in the presence of FruBM alone. **c** A lower dose of FruBM, which by itself had no effect on the reporter activity, was able to exert a strong repressor effect in the presence of TRF2-S. All experiments were conducted as triplicate and the mean ± SEM of relative luciferase activities are shown. Statistical differences were evaluated by one-way ANOVA followed by Bonferroni’s multiple comparison test. ***P < 0.001, **P < 0.01, *P < 0.05; ns not significant

TRF2-S activates transcription from a large number of promoters that lack a TATA-box^38–42^, whereas FruBM was recently shown to repress transcription from the promoter of *robo1*, a gene encoding a transmembrane receptor that governs the presence or absence of the ipsilateral neurite in mAL neurons^27^. We therefore examined the effect of TRF2-S on *robo1* transcription in the absence or presence of FruBM with reporter assays in S2 cells. We used a reporter expressing luciferase under the control of a 1.7 kb *robo1* promoter segment that carries a FruBM-binding site and thus is repressible by applying FruBM (Fig. 4a). We found that *Trf2-S* transfection significantly elevated the reporter activity (Fig. 4b). *fruBM* transfection (at 3 ng per 2 × 10^6^ cells) markedly repressed the reporter activity as expected (Fig. 4b). The FruBM effect to repress the reporter activity was dose-dependent and no discernible effect was obtained when one-third of the amount (i.e., 1 ng) was used for transfection (Fig. 4c). Remarkably, TRF2-S, which otherwise activates transcription, repressed transcription from the *robo1* promoter in the presence of a small amount of FruBM, even though this amount of FruBM alone was unable to repress transcription of the reporter (Fig. 4c). We conclude that TRF2-S exerts two contrasting effects on *robo1* transcription, i.e., activation and repression, dependent on the absence or presence of FruBM.

### TRF2-S binds to the *robo1* promoter

**Fig. 5:**
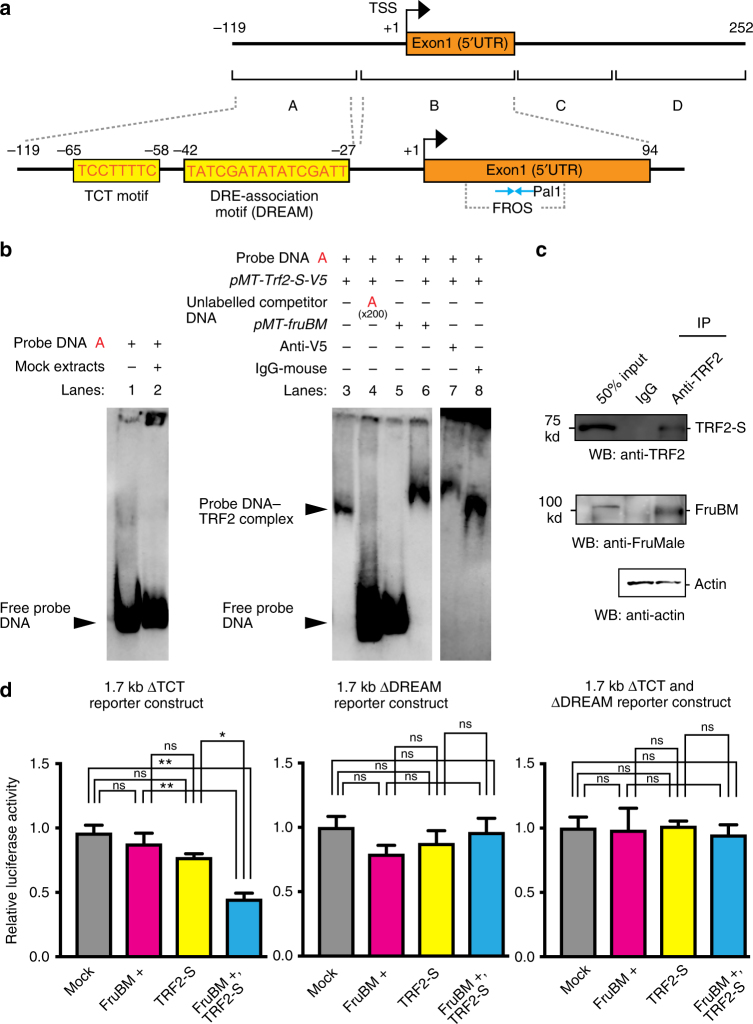
TRF2-S and FruBM interact with the *robo1* promoter. **a** Upper panel: the map of the *robo1* genomic region (upper line) and the positions of fragments A–D (lower line) used for electrophoretic mobility shift assays. Exon 1 is highlighted in orange. Transcription starts at the site and in the direction indicated by the arrow. Lower panel: the exon 1 and flanking regions with the TCT motif and DREAM are shown in an expanded scale. The location of FROS is indicated below exon 1. The two moieties of Pal1 as the core FruBM-binding motif are indicated by the blue arrows facing each other. The sequences composing the TCT motif and DREAM are indicated by red capitals. **b** Retarded migration of probe A was observed in the presence of S2 cell lysates expressing TRF2-S (TRF2-S[+] lysates, lane 3), but was not observed without lysates (lane 1) or with lysates expressing an empty vector (lane 2). The retarded band was diminished when an unlabeled probe A was added as a competitor (lane 4). Cell lysates expressing FruBM alone (FruBM[+] lysates, lane 5) did not yield any retarded band, whereas cell lysates expressing both TRF2-S and FruBM (TRF2-S[+]/FruBM[+] lysates, lane 6) did. The retarded band was supershifted when preincubated with a mouse anti-V5 antibody (lane 7), but not when preincubated with a mouse IgG antibody (lane 8). **c** Coimmunoprecipitation of TRF2-S and FruBM. Lysates from S2 cells co-transfected with *Trf2-S* and *fruBM* were subjected to immunoprecipitation with the anti-TRF2 antibody and analyzed by western blotting with the anti-TRF2 and anti-FruMale antibodies. Actin served as a loading control. **d** Luciferase reporter assays with the 1.7 kb *robo1* promoter region lacking TCT (∆TCT), DREAM (∆DREAM), or both (∆TCT and ∆DREAM) in S2 cells transfected with the empty vector (Mock), *fruBM* alone (FruBM), *Trf2-S* alone (TRF2-S), or *fruBM* plus *Trf2* (FruBM + TRF2-S). All experiments were conducted as triplicate and the mean ± SEM of relative luciferase activities are shown. Statistical differences were evaluated by one-way ANOVA followed by the Bonferroni’s multiple comparison test. **P < 0.01, *P < 0.05; ns not significant

The question arises as to whether a TRF2-S-containing complex binds to the *robo1* promoter to activate and repress its expression. To clarify this point, we conducted electrophoretic mobility shift assays with *robo1* promoter fragments in the presence and absence of TRF2-S, which was tagged with V5. A 101 bp fragment (the A fragment, Fig. 5a) derived from the region further upstream of FruBM-binding motif (FROS) revealed a retarded band, which disappeared in the presence of unlabeled fragment-A or exhibited a supershift upon the addition of an anti-V5 antibody that recognizes TRF2 (Fig. 5b). The mobility shift was not detected in the presence of FruBM, provided that TRF2-S was not co-transfected. Importantly, the A fragment contains a palindrome stretch, 5′-TATCGATA TATCGATT-3′, each moiety of which coincides with the binding consensus sequence DNA-replication element (DRE)-association motif (DREAM), to which DREF binds^43^. Furthermore, the polypyrimidine initiator motif TCT exists immediately 5′ to DREAM (Fig. 5a). Notably, TRF2 has been shown to associate with DREF^44^ and requires TCT for activating transcription^42^. This raises an intriguing possibility—namely, this palindrome may serve as the site for TRF2-*robo1* promoter interactions via DREF and TCT.

To evaluate the possibility that TCT and/or DREAM mediate the action of TRF2 to regulate *robo1* transcription, the effects of deleting either or both of these motifs from the 1.7 kb promoter fragment on the reporter activity were examined. When TCT was deleted (∆TCT), TRF2 was unable to increase the reporter activity, while it retained the ability to enhance the repressor action of FruBM (Fig. 5d). In contrast, loss of DREAM (∆DREAM) impaired the ability of TRF2 not only to increase the reporter activity but also to enhance the repressor activity of FruBM (Fig. 5d). As expected, deleting TCT and DREAM altogether (∆TCT and ∆DREAM) made the reporter unresponsive to TRF2 (Fig. 5d), as was ∆DREAM. We conclude that both TCT and DREAM are required in the *robo1* promoter for increasing the transcriptional activity in response to TRF2, whereas DREAM alone mediates the action of TRF2 to enhance the repressor activity of FruBM.

### TRF2-S associates with FruBM in vivo

Importantly, the A fragment has been shown not to contain a FruBM-binding site. An intriguing possibility is that the TRF2-containing complex interacts with the FruBM-containing complex upon binding to the respective target sequences, which are positioned close to each other on the genome (Fig. 5a). In an attempt to elucidate the molecular basis for this phenotypic similarity between *Trf2* and *fru*, we conducted immunoprecipitation assays to test the possibility that TRF2-S and FruBM interact with each other as components of a transcriptional machinery. To improve the power of the immunoprecipitation assays to detect protein–protein interactions, we conducted immunoprecipitation assays with lysates of S2 culture cells that had been transfected with tagged versions of *Trf2-S* and *fruBM*, confirming the association of TRF2-S and FruBM. We found that precipitates with an anti-TRF2 antibody contained not only TRF2 but also FruBM, as detected in western blotting by the antibodies that recognize the respective proteins (Fig. 5c). We suggest that the TRF2-S-containing complex interacts with the FruBM-containing complex in vivo, while each complex binds to a distinct site on the *robo1* promoter.

### Phenotypic outcome of *Trf2*, *fru*, and *robo1* interrelationships

To validate the importance of TRF2 actions on *robo1* transcription in establishing the FruBM-dependent sex-specific traits, we examined the effect of *robo1* knockdown on the *Trf2*-dependent induction of mAL neurons with the male-specific neurite. The increase in the proportion of single-cell mAL clones that lack the male-specific neurite by *Trf2* knockdown was, though statistically non-significant, ameliorated by the additional knockdown of *robo1* (Supplementary Fig. 4). We also examined whether *Trf2* knockdown results in the precocious wing switching, a change in the male courtship posture uniquely induced in male mutant flies (e.g., *robo1* ^*∆1*^ */robo* ^*∆3*^) whose *robo1* gene lacks a few nucleotides that compose the core binding motif for FruBM binding^27^. Strikingly, *Trf2* knockdown in *fru* ^*GAL4*^-expressing neurons phenocopied the *robo* ^*∆1*^ */robo* ^*∆3*^ mutants by exhibiting the precocious wing switching (Supplementary Fig. 5a, c). Furthermore, the effect of *Trf2* knockdown to induce the precocious wing switching was completely abrogated by simultaneous *robo1* knockdown (Supplementary Fig. 5b, c). These results are consistent with the idea that the disturbance of sex-specific traits by *Trf2* knockdown is, at least in part, expressed through the misexpression of *robo1* in *fru*-positive neurons including mAL neurons. It remains to be determined whether TRF2 regulates transcription of the FruM-target genes other than *robo1* and, if so, what aspects of sex-specific neurobehavioral traits are controlled by such mechanisms.

## Discussion

Although it is well recognized that FruBM plays a key role in establishing neural sexual dimorphisms, it remains largely unknown how the presence or absence of FruBM affects the transcription of target genes that induce neuronal sex differences^13^. By focusing on the *fru*-expressing neuronal cluster mAL, which displays sexual dimorphisms in the cell number, the contralateral neurite branching pattern, and the presence or absence of the ipsilateral neurite^28^, we successfully demonstrated that the core promoter regulator TRF2-S preferentially supports FruBM in the male-specific ipsilateral neurite formation, with only minor effects on two other aspects of mAL sexual dimorphisms. TRF2-S was found to assist FruBM by enhancing the repressor effect of FruBM on the promoter of *robo1*, the gene encoding a receptor for the axon guidance factor that inhibits the male-specific neurite formation in mAL neurons^27^. This finding was unexpected because TRF2 typically functions as a transcriptional activator rather than a repressor, being associated with the TATA-less promoter present in over 1000 genes in the genome^39–41^. Our results further demonstrated that TRF2-S is immunoprecipitated with the FruBM–protein complex, which was previously shown to contain chromatin regulators such as Bonus, HP1a, and HDAC1^25^. It is therefore plausible that TRF2-S is involved in chromatin organization in addition to its role as a core promoter recognition factor. In keeping with this idea, mouse mutants null for *Trf2* have been shown to exhibit a defect in chromatin condensation in early spermatids, leading to impaired spermiogenesis and male infertility^45^. Transcriptional repression by overexpressed TRF2 has been reported in TATA-dependent transcription, where TRF2 is postulated to absorb TFIIA^46^, a general transcription factor required for the initiation of transcription from the TATA-dependent promoter. Here we demonstrated that TRF2 acts on the TATA-box harboring the *robo1* promoter as a transcriptional activator in the absence of the masculinizer protein FruBM, or as a transcriptional repressor in its presence. Our analysis demonstrated that TRF2 may interact with the *robo1* promoter as mediated by the DREF-binding motif DREAM to enhance the repressor action of FruBM. Although DREF is generally considered to be a transcriptional activator, the DREF-containing complex turns into a transcriptional repressor when DREF recruits α-thalassemia and mental retardation X-linked syndrome (ATRX) to DREAM^47^.

Another important finding in this study was that, despite the striking effect of *Trf2* manipulation on the ipsilateral neurite formation, two other aspects of mAL sexual dimorphism, i.e., the different cell number and the branching pattern of the contralateral neurites, were only moderately affected by the manipulation of *Trf2*. Notably, all three aspects of the sexual dimorphisms of mAL are known to be FruBM-dependent^28^, implying that FruBM assembles different sets of transcriptional regulators to masculinize each of the three sex-specific traits. Therefore, the sex-type specification of a neuron likely proceeds under a layered system of transcriptional control that is spatially and temporally dynamic, and FruBM probably orchestrates this sophisticated network of transcription to render the brain circuitry distinct between the two sexes.

## Methods

### Fly strains

Flies were raised on cornmeal yeast medium at 25 °C. Canton S served as a wild-type control. The *Trf2* GS strain *GS9128/Binsinscy* (DGRC# 201543) and NP strain *NP6093/FM7c* (#105120) was obtained from the Kyoto stock center. The *UAS-HA-Trf2-S-V5* transformant lines were generated by two different methods, i.e., P-element-mediated random insertion and PhiC31-integrase-mediated site-directed integration. Fly resources from the Bloomington Drosophila Stock Center were used for the MARCM study. *UAS-Trf2 RNAi* (#10443 and #101318) and *UAS-robo1 RNAi* (#42578) fly lines were obtained from the Vienna Drosophila Resource Center. The fly line *(y hs-flp/+); FRTG13 UAS-mCD8::GFP; fru* ^*sat*^ *poxn-GAL4 (UAS-GFP)* was kindly provided by Dr. Ken-ichi Kimura.

### Behavioral assays

Flies were collected upon eclosion and reared individually under a 12 h:12 h light–dark cycle at 25 °C. About 5–7-day-old test males and 3–5-day-old Canton-S virgin females were used for the behavioral assays. To measure the CI, a male of each genotype and a Canton-S virgin female were paired in a small round chamber of 8 mm in diameter and 3 mm in height. The behaviors of paired flies were video recorded for 1 h. The video segment for the first 5 min after introducing the virgin female was used to calculate the CI, which was defined by the time spent by a test male for displaying courtship elements, including orientation, tapping, following, wing extension/vibration, and attempted copulation. For the WEI, only the time spent for vibrating/extending the wing was measured during the first 5 min after introducing the virgin female. The copulation success was measured based on whether the test male copulated within the 1-h observation period. Locomotion activity was estimated by quantifying the circular walk along the wall of a round chamber: the number of complete circles in both the clockwise and anti-clockwise directions made by a test male within 3 min after introduction to the chamber was counted and the values were transformed into the locomotion rate in mms^−1^. The wing switching index was estimated by the method as described in ref. ^27^. For every experiment, sample size was chosen based on preceding publications ensuring that it has adequate power to detect a meaningful differences across data sets.

### Dissection, immunohistochemistry, and imaging of tissues

For immunostaining, the central nervous system (CNS) of 3–5-day-old files was dissected in cold phosphate-buffered saline (PBS) with sharp forceps (Dumont #5). After dissection, the CNS was fixed in 4% paraformaldehyde for 1 h, followed by two 30 min washings in PBS with 0.2% Tween 20 (0.2% PBT). Then the CNS was kept in blocking buffer containing normal goat serum and 0.2% PBT for 1 h at room temperature. Immunostaining was performed using a rabbit anti-TRF2 antibody (a generous gift from James Kadonaga; at a dilution of 1:100), a guinea pig anti-FruMale antibody (a product of our laboratory; 1:500 dilution), a rabbit anti-GFP antibody (Invitrogen; 1:500 dilution), and a mouse anti-nc82 antibody (Developmental Studies Hybridoma Bank (DSHB), University of Iowa, Iowa City, IA; 1:50 dilution). CNS tissues were incubated with the primary antibody for 3 days, then subjected to six 10-min washings in 0.2% PBT. Secondary antibodies used were Alexa Fluor 488 anti-rabbit IgG and anti-guinea pig IgG, Alexa Fluor 546 anti-mouse IgG and anti-rat IgG, and Alexa Flour 647 anti-mouse IgG (Invitrogen, Carlsbad, CA; 1:200). The CNS was stained for 2 days with the secondary antibody and then washed for 30 min in 0.2% PBT twice. Finally, the CNS was mounted on a slide glass with 80% (v/v) glycerol in PBS. For the observation of germ cells (Supplementary Fig. 1), ovaries were dissected in PBS and blocked for 30 min in PBS containing 4% paraformaldehyde. The ovaries were washed three times in PBT, blocked for 1.5 h in a blocking buffer, and then incubated with a primary antibody at 4 °C overnight. The primary antibodies used were the rat anti-TRF2 (current study; 1:100 dilution) and rabbit anti-Vasa (d-260, Santa Cruz Biotechnology; 1:50 dilution) antibodies. Images were acquired with a Zeiss LSM 510 META confocal microscope using ZEISS LSM Image Browser software. All images were acquired with either 20× Plan-Apo/0.8 or 40× Plan-Apo/0.95 lenses. Images were acquired at a resolution of 512 μm × 512 μm with 1 μm intervals.

### Clonal analysis of mAL neurons

We used a *fru* ^*NP21*^ *-GAL4* line to label mAL neurons. The somatic clones were produced using the MARCM method^35^. The flies with the genotype *y hs-flp/Y or w; FRTG13 UAS-mCD8::GFP/FRTG13 tub-Gal80; fru* ^*NP21*^ *,UAS-Dcr2/+* were used as the control males or females. The genotype of flies used in clonal *Trf2* knockdown experiments was *y hs-flp/Y* (for males) or *w* (females); *FRTG13 UAS-mCD8::GFP/FRTG13 tub-Gal80; fru* ^*NP21*^ */UAS-Trf2 RNAi* (VDRC# 10443). For the production of neuroblast clones in mAL neurons, embryos at 0–24 h after egg-laying (AEL) were heat shocked at 37 °C in a water bath for 1 h. For the production of single-cell clones of mAL neurons, larvae at 4–6 days AEL were heat shocked at 37 °C for 1 h. Flies to be tested were reared at 29 °C after the heat shock in order to enhance the expression of transgenes.

### S2 cell culture

*Drosophila* S2 cells were cultured in Schneider’s *Drosophila* medium (Gibco, 21720-024) supplemented with 10% heat-inactivated fetal bovine serum (HyClone, SH30071.03) and 1% penicillin–streptomycin solution (Gibco, 15140613) to prevent bacterial contamination.

### Western blot assays

S2 cells were disrupted by incubating with a lysis buffer (50 mM HEPES, pH 7.5, 300 mM NaCl, 50 μM ZnSO_4_, 10 mM NaF, 0.4% Nonidet P-40 (NP40), and cOmplete Protease Inhibitor (Roche)) for 1 h at 4 °C. Lysates were prepared by centrifugation (15,000 rpm for 15 min) and denatured in the sample buffer. Samples were fractionated using 7.5% SDS–PAGE gel (e-PAGEL, E-T 7.5L) at 100 mV. Then blots were transferred to PVDF membranes (Life Technologies, IB401001), and reacted with rat anti-TRF2 (1:100; present study), rabbit anti-Fru Male (1:500)^20^ or mouse anti-α-actin (1:500; Abcam, ab3280) overnight, and subsequently with a horseradish peroxidase (HRP)-conjugated anti-rat, rabbit, or mouse IgG antibody (1:3000; Sigma) for 3 h. Pierce Western Blotting Substrate Plus (Thermo Scientific, NCI32132) was used according to the manufacturer’s instructions to detect chemiluminescence. Fluorescent images were obtained using an ImageQuant LAS 4000 system (Fujifilm).

### TRF2-S expression vectors

TRF2-S was cloned from *Drosophila* embryo complimentary DNAs and expressed with an N-terminal HA tag and C-terminal V5 tag in S2 cells using either the pact or pMT/V5-His C expression vector (Invitrogen). The sequence encoding HA-Trf2-S-V5-His was cloned independently in the pUAST and pJFRC81 vectors. These vectors were used to generate flies expressing *UAS-HA-Trf2-S-V5-His* by standard injection protocols.

### Coimmunoprecipitation assays

In the coimmunoprecipitation assays for TRF2-S and FruBM, both proteins were overexpressed in S2 cells. About 1 μg of each of the pMT-HA-Trf2-S-V5-His and pMT-FLAG-fruBM plasmid vectors was transfected into S2 cells (1 × 10^7^ cells) using FugeneHD (Roche Diagnostics, Indianapolis, IN), and protein expression was induced by addition of copper sulfate. Lysates were prepared by homogenizing in a cold lysis buffer (50 mM HEPES, pH 7.5, 100 mM NaCl, 50 μM ZnSO_4_, 10 mM NaF, 0.2% NP40, and cOmplete Protease Inhibitor (Roche)) for 1 h at 4 °C, then incubated with rat IgG (Invitrogen, I0700) or the rat anti-TRF2 antibody (present study) in the aforementioned lysis buffer for 3 h at 4 °C. The immunocomplexes were precipitated using Dynabeads Protein G (Invitrogen, 10004D) according to the manufacturer’s instructions. Finally, the immunocomplexes were analyzed by western blotting with a primary antibody, i.e., rabbit anti-FruMale (1:500)^20^ and rat anti-TRF2 (1:100; present study), and, as a secondary antibody, with HRP-conjugated anti-rabbit or anti-rat IgG antibody (1:3000; Sigma).

### Reporter assays

Reporter assays were carried out with the *robo1* promoter luciferase reporter as described previously^27^. The pGL3-promoter vector carrying a 1.7 kb *robo1* promoter fragment was used as a reporter construct. The pRL-TK *Renilla* luciferase vector (Promega) served as an internal control. About 100 ng of a reporter construct and 10 ng of an internal control were co-transfected into S2 cells (2 × 10^6^ cells) with either pact-FLAG-fruBM and/or pact-HA-Trf2-S-V5-His and pact-MCS^25^ using FugeneHD (Roche Diagnostics, Indianapolis, IN). Cells were lysed after 36–48 h of transfection with a passive lysis buffer (Promega), and luciferase activity was measured using a Dual-Luciferase Assay System (Promega). To standardize the transfection efficiency, the reporter luciferase activity of each sample was normalized to the corresponding control *Renilla* luciferase activity: the relative luciferase activity of a reporter construct was calculated relative to that carrying only an empty pact-MCS plasmid. All experiments were carried out in triplicate; the relative luciferase activities are shown as the mean ± SEM.

### Electrophoretic mobility shift assays

pMT-FLAG-fruBM and pMT-HA-Trf2-S-V5-His were transfected together or individually into S2 cells, and the expression of proteins was induced by the addition of copper sulfate. Electrophoretic mobility shift assays experiments were carried out using a DIG Gel Shift Kit (Roche Diagnostics). Binding reactions were established as follows: 1× binding buffer (20 mM Hepes, pH 7.5, 10 mM (NH_4_)_2_SO_4_, 0.2% Tween 20, 30 mM KCl, and 50 μM ZnSO_4_), 0.1 μg µl^−1^ poly [d(I-C)], 5 ng µl^−1^ poly l-lysine, 5 µl of nuclear extract, and 1 ng of a digoxigenin (DIG)-labeled probe in a final volume of 20 µl. A 25- to 200-fold excess of unlabeled double-stranded oligonucleotide was included in an experiment to detect competing reactions. Reactants were incubated at room temperature for 20 min. For antibody-binding assays, the mouse anti-V5 antibody was added to the sample solution and incubated for 15 min prior to binding assays. Binding reactions were resolved on 5% polyacrylamide gels and transferred to a nylon membrane (Life Technologies, IB801001). The membrane was then UV cross-linked, blocked and probed with anti-DIG antibodies. DIG-labeled DNA–protein complexes were detected by chemiluminescence using an ImageQuant LAS 4000 system (Fujifilm).

### Midline crossing score analysis

The MCS was calculated as described previously^25^. Briefly, stacked images of each sample were summed up using ImageJ, and a circle of 8–10 μm (marked as “a” in panel f of Fig. 3) was drawn on the resultant image so that the circle was centered at the prothoracic midline, where trans-midline axons are expected to run in males. The fluorescent intensity within the circle marked “a” was measured to quantify the level of midline crossing by fibers. Similarly, the fiber tracts locating lateral to the midline were quantified within circles “b1” and “b2” for the fluorescent intensity. To normalize for the background fluorescent level, the areas with no fibers delineated by circles “c1” and “c2” were also sampled. The MCS was calculated as: MCS = [a − (c1 + c2)/2]/[(b1 + b2)/2].

### Antibody production

To generate the anti-TRF2 antibody, which recognizes a stretch of 14 amino acids (KLQNKRPRYNDPGT) in the C-terminus of TRF2, a 14-amino-acid peptide was synthesized, and rat antiserum against this peptide was raised and affinity-purified.

### Statistical analysis

Statistical analyses were done by GraphPad Prism 7.0b software.

### Data availability

The data sets generated during and/or analyzed during the current study are available from the corresponding author on reasonable request.

## Electronic supplementary material

Supplementary information

Peer Review
