# Supplementary material for: The core-promoter factor TRF2 mediates a Fruitless action to masculinize neurobehavioral traits in Drosophila
Source: Nat Commun. 2017 Nov 14;8:1480. doi: 10.1038/s41467-017-01623-z (PMC5684138; doi:10.1038/s41467-017-01623-z)
Supplement: Supplementary file 1 — Supplementary information [file 41467_2017_1623_MOESM1_ESM.pdf]

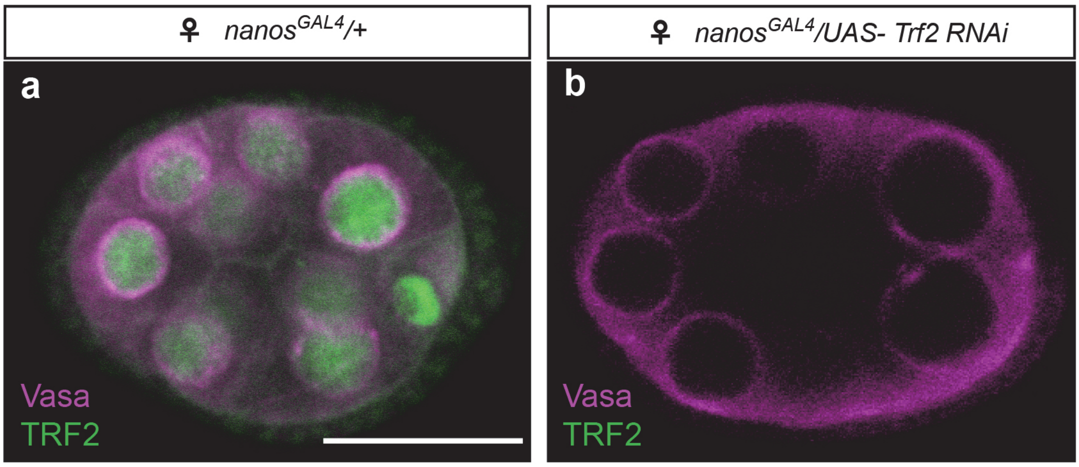

Supplementary Fig. 1| Test for the efficacy of *Trf2* knockdown with the *Trf2 RNAi* transgene. The egg chambers were stained for TRF2 (green) and the germ cell marker Vasa (magenta) with the respective antibodies in *nanos-GAL4* control flies (**a**) and a fly expressing *UAS-Trf2 RNAi* as driven by *nanos-GAL4* (**b**). The intensity of TRF2 immunoreactivity in the nurse cell nuclei was reduced upon *Trf2* knockdown. Scale bar: 20 µm.

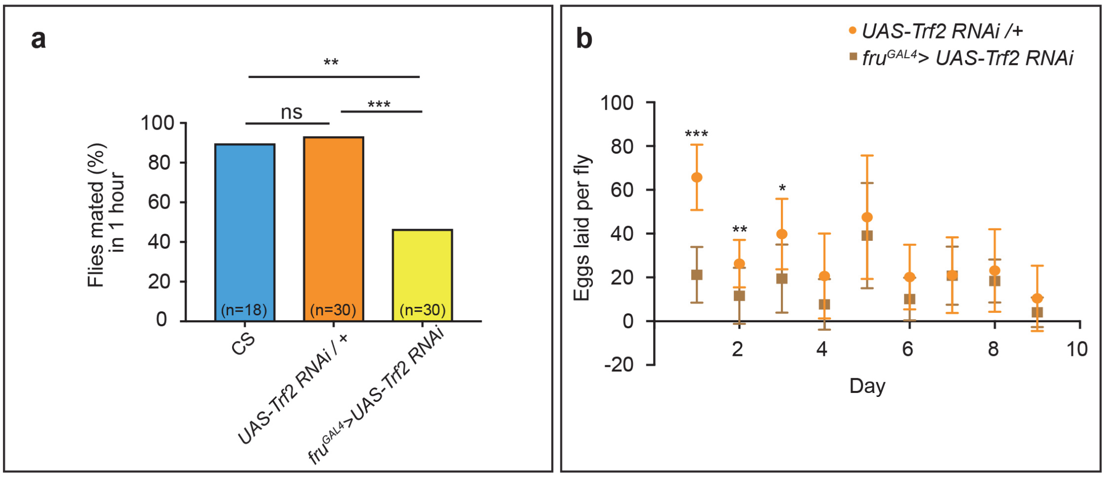

Supplementary Fig. 2| Effects of *Trf2* knockdown on female-specific traits. The mating success rate (**a**) and the number of eggs a female laid per day (**b**) were compared between control flies and the flies with *Trf2* knockdown. In evaluating the mating success rate (**a**), female and male pairs were observed in a round chamber (60 mm in diameter and 15 mm in height) for 1 h to calculate the percentage of mated pairs. The numbers of observed pairs are indicated in parentheses on the bars. The statistical differences were evaluated by the χ^2 test. ***P<0.001, **P<0.01; ns: non-significant. In quantifying the egg deposition (**b**), a female was mated with a male by placing them in a small vial with food for 3 h, and then the female was transferred to a new vial, allowing her to lay eggs. Thereafter, the female was transferred to a new vial every day and the number of eggs laid in the old vial was counted. The mean ± SD values are indicated. The statistical differences were evaluated by the Student's *t*-test, ***P<0.001, **P<0.01, *P<0.05.

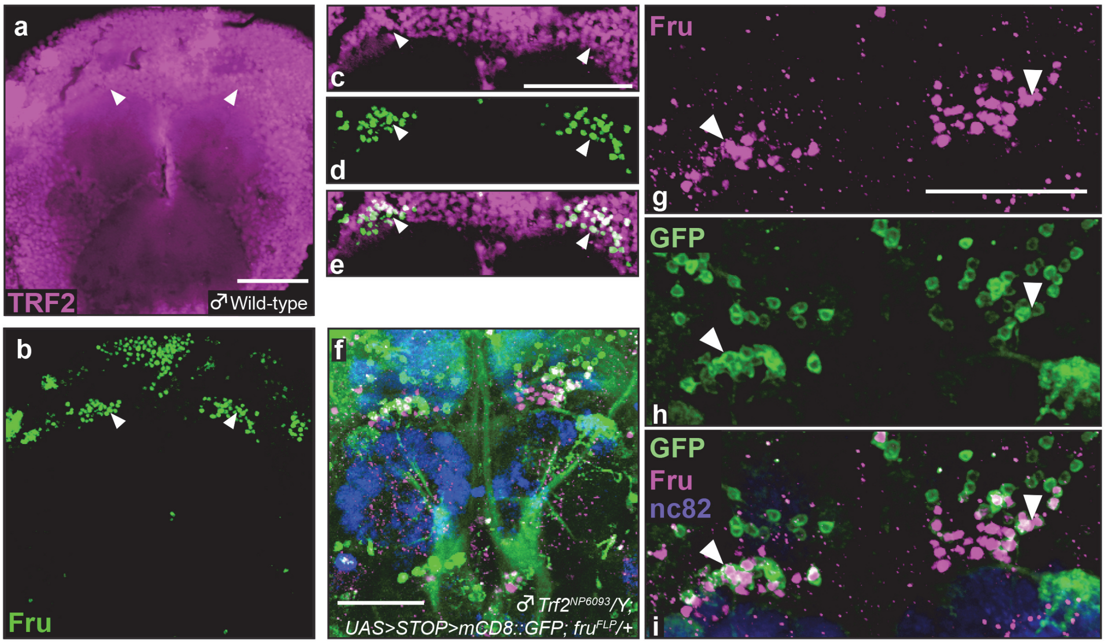

Supplementary Fig. 3| mAL neurons express *Trf2*. a-e, A wild-type male brain doubly stained with the anti-TRF2 antibody (**a, c**: magenta) and the anti-FruM antibody (**b, d**: green). (**e**) represents a merged image of (**c**) and (**d**). The mAL cluster is indicated by arrowheads. **g-i**, A male brain triply stained with an anti-GFP antibody that labels cells positive for *NP6093-GAL4* (*Trf2^{NP6093}*), an enhancer trap line for *Trf2* (**h**, **i**: green), an anti-FruBM antibody (**g**, **i**: magenta) and the monoclonal antibody nc82 (**f**, **i**: blue), which labels all arborizations. Enlarged views of the mAL cluster (arrowheads) triply stained are shown in (**g-i**). Cells doubly positive for *Trf2* and *fru* were labeled by the intersection technique with *fru^{FLP}* and *UAS>STOP>mCD8::GFP*. Scale bar: 50 µm.

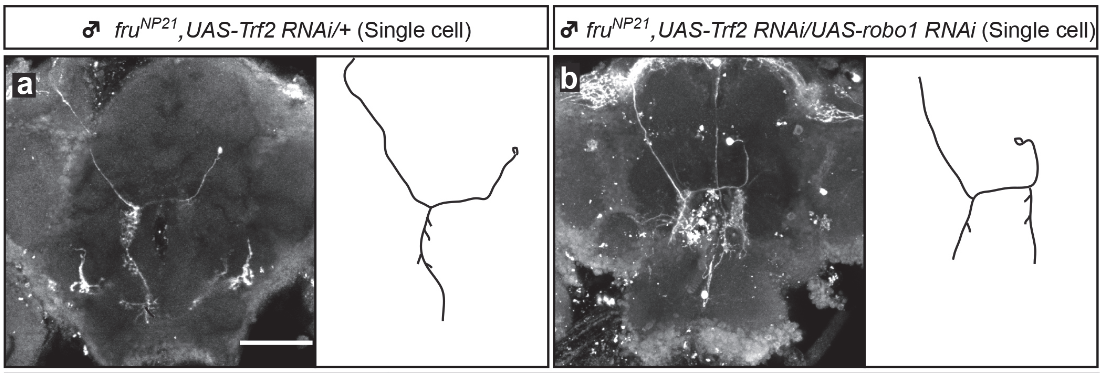

c Genotype	Single mAL neurons	
	With ipsilateral neurite (%)	Without ipsilateral neurite (%)
♂ fru^{NP21},*UAS-Trf2 RNAi*/+ (n=23)	34.78	65.22
♂ fru^{NP21},*UAS-Trf2 RNAi*/ *UAS-robo1 RNAi* (n=35)	57.14	42.86

Supplementary Fig. 4| *robo1* knockdown impedes the *Trf2*-knockdown effect to reduce the proportion of mAL neurons carrying the ipsilateral neurite. a,**b**, Examples of single-cell clones of mAL neurons without (**a**) or with (**b**) the ipsilateral neurite. The genotype of flies is indicated at the top. **c**, The proportion of mAL neurons with and without the ipsilateral neurite in flies subjected to *Trf2* knockdown is compared between the fly group with additional *robo1* knockdown and that without *robo1* knockdown. The frequency of obtaining neurons with the ipsilateral neurite increased after *robo1* knockdown in a statistically non-significant level by the χ^2 test. Scale bar: 50 µm.

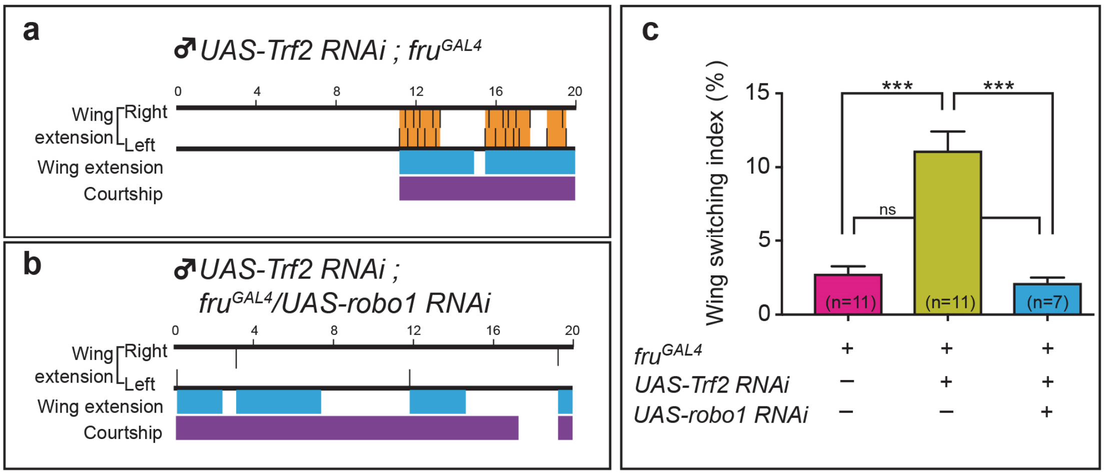

Supplementary Fig. 5| Precocious wing-switching phenotype induced by *Trf2* knockdown and its rescue by additional *robo1* knockdown in mAL neurons. a, **b**, Examples of ethograms showing wing extension and courtship activities in males of the indicated genotype. The time elapsed since the start of observation is shown on the top. The period during which the fly displayed precocious wing-switching (orange bar), wing extension (blue bar), or any courtship actions (purple bar) is shown. Vertical lines above the blue bars indicate the time at which the fly switched the wing to be extended from the left wing to the right wing (right) and vice versa (left). **c**, The wing switch index for three genotypic groups. *Trf2* knockdown increased the precocious wing-switching, which was suppressed by additional *robo1* knockdown. Error bars represent SEM. Statistical differences were evaluated by one-way ANOVA followed by Tukey's multiple comparison test. ****P*<0.001; ns: not significant.
