# Supplementary material for: The core-promoter factor TRF2 mediates a Fruitless action to masculinize neurobehavioral traits in Drosophila
Source: Nat Commun. 2017 Nov 14;8:1480. doi: 10.1038/s41467-017-01623-z (PMC5684138; doi:10.1038/s41467-017-01623-z)
Supplement: Supplementary file 2 — Peer Review [file 41467_2017_1623_MOESM2_ESM.pdf]

Reviewers' comments:

Reviewer #1 (Remarks to the Author):

The current paper addresses a very important issue of how fruitless at the molecular level masculinizes the otherwise female neurons in fruit fly. The authors use variety of molecular biology and biochemical tools to demonstrate that TRF2-S binds to FruBM protein to suppress robo1 gene transcription.
I think authors deserve a good credit for performing a genetic screen and identifying the target and verifying it at the molecular level. However when authors try to argue that TRF2-S is involved in masculinization of neurite morphology as well as in behavioral modification of courtship behavior I think there are some criticisms that need to be addressed.

1. Authors need to show that TRF2-S is expressed in fruitless neurons and specifically in mAL neurons that show neurite morphology changes in TRF2-S knock-down experiments. This is important to address the issue if TRF2-S in wild type animals is acting on the neurons to change their morphology.
2. Authors claim that TRF2-S protein changes female neurons into male like neurons and their claim is based on the knock-down and overexpression studies in mAL and other neurons. However some of the male like morphology of the neurons that overexpress or downregulate TRF2-S expression is not affected. The study begs the question what is different in those neurons. Is it that TRF2-S is not expressed in those neurons or they don’t express robo1? Again immunohistochemistry may shed some light on this issue.
3. Authors claim that TRF2-S downregulation affects courtship behavior in males. Although this is a correct statement one could easily imagine disruption of courtship ritual by any general transcription factor that changes cellular morphology. If authors claim that TRF2-S is masculinizing in the presence of fruBM and feminizing in it’s absence than one would expect that sex specific behavioral phenotypes should be affected also in females. If it turns out to be that TRF2-S knock-down masculinizes female behaviors i.e. reduces receptivity and egg laying it would raise the impact of the paper significantly. Even more interesting would be if females show male like courtship behaviors towards other females.
4. Finally we get a snapshot of how selected neurons are affected by TRF2-S knock-down but what is the global picture? How does fruitless neurons are affected in terms of their overall numbers and cluster distribution?

If authors address these issues I believe paper will improve significantly.

Reviewer #2 (Remarks to the Author):

Chowdhury et al performed a genetic screen to identify proteins that interact with Drosophila male Fru, a protein important for establishing male courtship behavior. The genetic screen is based on the observation that GMR-Gal4-driven overexpression of a common isoform of Fru (FruB) in the eye causes a rough eye phenotype, with disordered bristles. The authors state they cannot use the male FruB isoform, as overexpression causes lethality. To identify enhancers and suppressors of this eye phenotype they screen a panel of GS P elements that will cause overexpression of the adjacent gene driven by GMR-Gal4, in the genetic background with the UAS-FruB transgene. They find that a P element inserted adjacent to TRF-2 can suppress the eye phenotype and further prove this is due to overexpression of TRF-2 by showing suppression using an engineered UAS-TRF-2 transgene. Based on this they examine behavior and neuron morphology. They show reduction of TRF-2 in Fru male neurons causes courtship defects. They examine a set of mAL cluster neurons that have three sexually dimorphic features and find that reduction of TRF-2 only impacts one feature, a neurite outgrowth

phenotype. They also examine a region in the ventral nerve cord and find that loss of TRF-2 reduced male-type midline crossing and gain of TRF-2 restores male-type midline crossing in a fru mutant background. They use a tissue culture transfection system to examine TRF-2 and FruBM roles on transcription of an upstream DNA region from robo, a FruBM target they previously identified. They find expression of TRF-2 alone increased transcription, FruBM represses and FruBM/TRF-2 together repress. They use gel shift assays to test if FruBM and/or TRF-2 directly bind this DNA and co-immunoprecipitation assays to test for protein interactions.

Criticisms:
The basis of identifying TRF-2 as a FruB interaction partner is suppression of a rough eye phenotype when FruB is overexpressed. How specific is this suppression, given TRF-2 is a common core promoter regulator? Any protein that when overexpressed reduced FruB activity is expected to reduce the rough eye phenotype, including any protein that can directly or indirectly negatively regulate core promoter activity, like TRF-2 can, as stated by the authors in the discussion. The statement that overexpression of TRF-2 does not reduce a tramtrack rough eye phenotype does not really get at specificity, given that tramtrack may function differently than FruB, even though both are BTB proteins. Thus, their major finding is that a core promoter regulator genetically interacts with a transcription factor.

The morphological phenotypes observed due to knockdown of TRF-2 are difficult to interpret with respect to FruBM function, given the pleiotropic nature of TRF-2. The neurite phenotypes could be due to the activities of other transcription factors that interact with TRF-2 in this population of neurons, including, for example, downstream target genes of FruBM that are transcription factors.

Their approach to show specificity for courtship is also not convincing, as they perform the knockdown of TRF-2 only in Fru neurons. Are Fru neurons required for locomotor behaviors?

In the tissue culture assay, the authors find that adding TRF-2 increased expression of a reporter through a 1.7KB upstream region of robo. Addition of FruBM reduced expression. In both these cases the differences were modest, though significant. The reduction by FruBM required a large amount of DNA to be transfected, and thus FruBM may be acting to sequester factors needed for transcription in a non-specific manner. In panel 4C there is no significant difference from mock, when FruBM or FruBM and TRF-2 are transfected. The modest significant differences are present only in comparison to TRF-2 transfection conditions, where there was a significant increase in reporter activity. There are many possible interpretations of this result.

Strong data supporting direct binding interactions on this DNA element are thus critical, which the authors attempt by gel shift assays. The gel shift assay does not have sharp bands, but is very smeary. The supershift in lane 5 after addition of FruBM is not very convincing. How repeatable is this result? Also, the authors do not have a lane with FruBM and not TRF-2. Interference by anti-TRF-2 antibody is not convincing either, given that addition of an antibody can interfere in non-specific ways. The proteins they made in the extracts were tagged. Did they try antibodies against the epitope tags? A supershift would be much more convincing.

General comments:

Some of the language in the paper was redundant, especially topics in the introduction and beginning of results.

Lines 158-160 are very confusing.

Did the authors only look at 4 females brains? Line 170. That is a small N for this type of experiment.

Reviewer #3 (Remarks to the Author):

A major goal in behavioral neurogenetics is to establish causal links between the actions of developmental regulatory genes, the development of neurons and circuits, and the generation of innate behaviors. In Drosophila, male-specific products of the fruitless gene (fruM) are critical in building the neural potential for male courtship behavior. Fru encodes a BTB-containing transcription factor. How exactly fruM specifies male behavior has only recently started to become unraveled. The Yamamoto group has been a pioneer in this regard. Focusing on the sex-specific development of a defined fruM-expressing neural class, the mAL neurons, the Yamamoto group recently identified the robo1 gene as a direct transcriptional target of fruM (specifically the B isoform of fruM: fruBM). They demonstrated that fruBM represses the expression of robo1 in the mAL neurons, thereby allowing the development of a male-specific ipsilateral neurite. In the absence of fruBM, as in females, the mAL neurons fail to develop the male-specific neurite due to the expression of robo1.

The manuscript by Chowdhury et al is a substantial step forward in our understanding of the molecular mechanisms by which fruBM regulates robo1 expression. In this study, Chowdhury et al submit that fruBM regulates robo1 through the core promoter factor, Trf2. A combination of behavioral, neuroanatomical and biochemical experiments suggest that Trf2 promotes both male and female development based on the presence or absence of fruBM. More specifically, the authors propose an intriguing model wherein Trf2 promotes robo1 expression in females, whereas in males, the presence of fruBM ‘switches’ Trf2 into repressor, thereby shutting down robo1 expression. They propose that these interactions occur in cis at the robo1.

The work described in Chowdhury et al is well worth publication, as their conclusions are a big leap in our understanding of the molecular mechanisms by which fruM specifies sex-specific development. That said, the authors do not provide enough experimental support for their claims, and I highly recommend that they include additional experiments before publication. In particular, the manuscript lacks any in vivo genetic support for the interactions between fruBM, Trf2 and robo1. My concerns are enumerated below.

1. The authors claim that Trf2 activates robo1 expression in females, whereas in males, the presence of FruBM converts Trf2 into a repressor, thereby turning robo1 expression off. These molecular interactions account (at least in part) for the sex-specific development of the ipsilateral mAL neurite.

As the paper stands, the authors provide no in vivo genetic support for the link between FruBM, Trf2 and robo1. I recommend that the authors do include genetic experiments that test the proposed interactions between FruBM, Trf2 and robo1. For example:

(a) If Trf2 and FruBM repress robo1 expression in males, then the loss of the male-specific ipsilateral mAL neurite in Trf2 knockdown males should be dependent upon the gain of robo1 expression. The authors could test this by asking if the knockdown of robo1 rescues Trf2 mutant neuroanatomy in males.

(b) Based on their biochemical data, the authors claim that FruBM represses robo1 through Trf2. The idea is that FruBM ‘switches’ Trf2 from being an activator to being a repressor. If this were true, then the authors should be able to rescue the loss of the male-specific ipsilateral mAL neurite in FruBM mutants by knocking down Trf2 function. For instance, the Yamamoto group published a robo1 mutant

that lacks the putative FruBM binding site. This mutant produces “dwarf” male-specific neurites. Is this suppressed by the concomitant loss of Trf2?

(c) The authors may also be able to over-express fruBM in the mAL neurons of females, which would presumably cause a gain of the male-specific neurite. They can then ask whether the resulting phenotype is dependent upon the function of Trf2.

2. The authors use luciferase reporter assays to provide in vitro evidence that Trf2 is an activator by itself, but a repressor in the presence of FruBM. As it stands now, this claim is established with just a single experiment. I recommend that the authors do additional experiments with mutant constructs to validate their results. For example:

(a) Luciferase reporter expression is repressed when Trf2 is co-transfected with low amounts of FruBM. Based on this result, the authors suggest that Trf2 becomes a repressor in the presence of fruBM. This is a substantial claim, and it deserves to be buttressed with additional experiments. For instance, the authors could repeat this experiment using a mutant construct in which the putative fruBM binding site has been mutated. If what they are claiming is true, then reporter expression should increase above the baseline, as Trf2 would activate transcription.

(b) The repression observed when co-transfecting FruBM and Trf2 should be dependent upon the putative Trf2 binding site. This is testable: The authors could repeat the experiment in Fig 4C with the Trf2 binding site mutated; this should cause a loss of repression, restoring reporter expression to baseline levels.

3. Wing switching. The authors should provide genetic evidence for the interactions between fruBM, Trf2 and robo1 in behavior. Previously, the Yamamoto group (Ito et al., 2016) was able to rescue the wing switching phenotype of robo1-delta1 mutants (these mutants carry mutations in the putative fruBM binding site, as noted above) by expressing an RNAi against robo1 (using 43D01-Gal4). This supported the notion that the delta1 mutation caused an upregulation of robo1 expression.

Based on the data presented in the current manuscript, one might predict that the knockdown of Trf2 would also rescue the wing switching phenotype of robo1-delta mutants (b/c the gain of robo1 expression should be dependent upon Trf2.): 43D01-Gal4 -> UAS-Trf2-RNAi, robo1-delta1/+ versus robo1-delta1. Notably, if this experiment failed to rescue, it may add to the authors’ idea that fruBM regulates target gene expression (including robo1) in a variety of ways.

Also, the authors should tell us to what extent the loss of Trf2 in males phenocopies robo1-delta1 mutants.

4. Does Trf2 contribute to female behavior? The authors provide evidence that Trf2 promotes male behavior and neuroanatomy in the presence of FruM. They also provide evidence that Trf2 feminizes neuroanatomy, but leave female behavior out. The authors should attempt to knockdown Trf2 in females using fruGal4, for example, and asking whether or not female receptivity or egg laying is affected.

Point-by-point replies to the reviewers' comments

Reviewer #1 (Remarks to the Author):

The current paper addresses a very important issue of how fruitless at the molecular level masculinizes the otherwise female neurons in fruit fly. The authors use variety of molecular biology and biochemical tools to demonstrate that TRF2-S binds to FruBM protein to suppress robo1 gene transcription.
I think authors deserve a good credit for performing a genetic screen and identifying the target and verifying it at the molecular level. However when authors try to argue that TRF2-S is involved in masculinization of neurite morphology as well as in behavioral modification of courtship behavior I think there are some criticisms that need to be addressed.

Q1. Authors need to show that TRF2-S is expressed in fruitless neurons and specifically in mAL neurons that show neurite morphology changes in TRF2-S knock-down experiments. This is important to address the issue if TRF2-S in wild type animals is acting on the neurons to change their morphology.
A1. In the revised manuscript, we have shown that TRF2 is expressed in mAL neurons by use of a *Trf2* enhancer-trap reporter and by double staining of the brain with the anti-TRF2 and anti-FruM antibodies (Supplementary Fig. 3; lines 141–142, page 10).

Q2. Authors claim that TRF2-S protein changes female neurons into male like neurons and their claim is based on the knock-down and overexpression studies in mAL and other neurons. However some of the male like morphology of the neurons that overexpress or downregulate TRF2-S expression is not affected. The study begs the question what is different in those neurons. Is it that TRF2-S is not expressed in those neurons or they don't express robo1? Again immunohistochemistry may shed some light on this issue.
A2. As mentioned above, TRF2 is expressed in all cells within the mAL cluster. In our previous work (Ito et al., 2012, Cell 149, 1327-1338), we demonstrated that sexual transformation from the male-type to the female-type of single mAL neurons in the male brain follows an all-or-none rule such that, as normal FruM function is reduced, more cells convert from the male-type to female-type; as a result, in FruM null males, all mAL neurons turn into female-type (Kimura et al., 2005, Nature 438, 229-233), whereas, in hypomorphic mutants, the female-type and male-type neurons coexist in the mAL cluster and the proportion of each depends on the level of residual *fru* gene function (Ito et al., ditto). The manipulations of *Trf2* or *robo1* employed in the current experiment were intended to simulate the *fru* hypomorphic conditions, and thus the presence of male-type neurons (i.e., cells not sexually transformed) together with female-type neurons (i.e., cells sexually transformed) was an expected result.

Q3. Authors claim that TRF2-S downregulation affects courtship behavior in males. Although this is a correct statement one could easily imagine disruption of courtship ritual by any general transcription factor that changes cellular morphology. If authors claim that TRF2-S is masculinizing in the presence of fruBM and feminizing in it's absence than one would expect that sex specific behavioral phenotypes should be

affected also in females. If it turns out to be that TRF2-S knock-down masculinizes female behaviors i.e. reduces receptivity and egg laying it would raise the impact of the paper significantly. Even more interesting would be if females show male like courtship behaviors towards other females.

A3. In our additional experiment, we found that *Trf2* knockdown in *fru*^{GAL4}-expressing neurons in females significantly reduced receptivity and egg deposition (lines 129 –132, page 9 and Supplementary Fig. 2).

Q4. Finally we get a snapshot of how selected neurons are affected by TRF2-S knock-down but what is the global picture? How does fruitless neurons are affected in terms of their overall numbers and cluster distribution?

A4. *Trf2* knockdown with the *fru*^{GAL4} driver had only moderate effects on the total number of *fru*^{GAL4}-positive neurons in the brain: 1865.33 ± 352.65 cells (control) vs. 1714 ± 151.26 cells (*Trf2* knockdown) in females and 2088 ± 282.13 cells (control) vs. 2006 ± 238.7 cells (*Trf2* knockdown) in males (n=3 for each fly group). The entire *fru*-circuitry in flies with Trf2 knockdown visualized by the combination of GFPN plus *fru*^{GAL4} was, in practice, indistinguishable from that in control flies, as shown in the pictures attached below. However, clonal labeling as done in the current study will reveal changes in neural structures induced by *Trf2*-knockdown in *fru* neurons other than mAL.

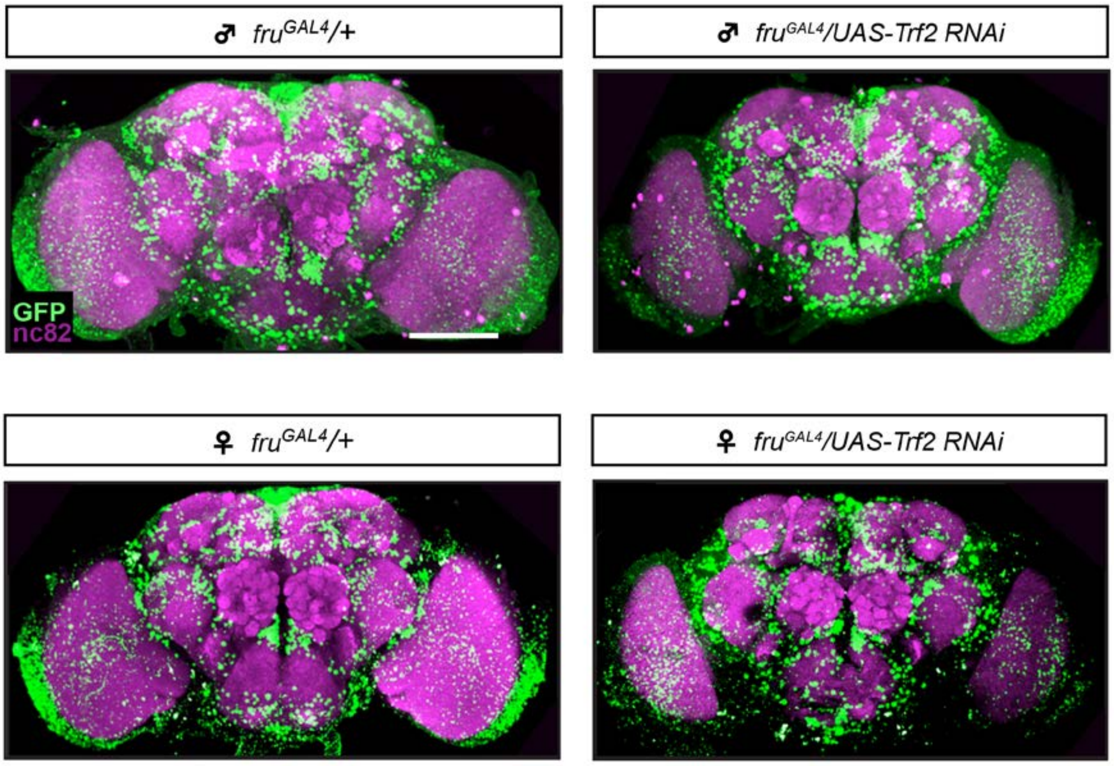

Reviewer #2 (Remarks to the Author):

Chowdhury et al performed a genetic screen to identify proteins that interact with Drosophila male Fru, a protein important for establishing male courtship behavior. The genetic screen is based on the observation that GMR-Gal4-driven overexpression of a common isoform of Fru (FruB) in the eye causes a rough eye phenotype, with disordered bristles. The authors state they cannot use the male FruB isoform, as overexpression causes lethality. To identify enhancers and suppressors of this eye phenotype they screen a panel of GS P elements that will cause overexpression of the adjacent gene driven by GMR-Gal4, in the genetic background with the UAS-FruB transgene. They find that a P element inserted adjacent to TRF-2 can suppress the eye phenotype and further prove this is due to overexpression of TRF-2 by showing suppression using an engineered UAS-TRF-2 transgene. Based on this they examine behavior and neuron morphology. They show reduction of TRF-2 in Fru male neurons causes courtship defects. They examine a set of mAL cluster neurons that have three sexually dimorphic features and find that reduction of TRF-2 only impacts one feature, a neurite outgrowth phenotype. They also examine a region in the ventral nerve cord and find that loss of TRF-2 reduced male-type midline crossing and gain of TRF-2 restores male-type midline crossing in a fru mutant background. They use a tissue culture transfection system to examine TRF-2 and FruBM roles on transcription of an upstream DNA region from robo, a FruBM target they previously identified. They find expression of TRF-2 alone increased transcription, FruBM represses and FruBM/TRF-2 together repress. They use gel shift assays to test if FruBM and/or TRF-2 directly bind this DNA and co-immunoprecipitation assays to test for protein interactions.

Criticisms:
Q5. The basis of identifying TRF-2 as a FruB interaction partner is suppression of a rough eye phenotype when FruB is overexpressed. How specific is this suppression, given TRF-2 is a common core promoter regulator? Any protein that when overexpressed reduced FruB activity is expected to reduce the rough eye phenotype, including any protein that can directly or indirectly negatively regulate core promoter activity, like TRF-2 can, as stated by the authors in the discussion. The statement that overexpression of TRF-2 does not reduce a tramtrack rough eye phenotype does not really get at specificity, given that tramtrack may function differently than FruB, even though both are BTB proteins. Thus, their major finding is that a core promoter regulator genetically interacts with a transcription factor.
A5. The reviewer appears to have some doubt about the validity of our screens with the compound eye system for isolating genes that act together with *fru* in neural sexual differentiation. Our subsequent analysis of *Trf2* functions in *fru*-expressing mAL neurons proved the unique roles of *Trf2* in the sex-type specification of these neurons, and this fact itself would be the best rebuttal to these particular criticisms.

Q6. The morphological phenotypes observed due to knockdown of TRF-2 are difficult to interpret with respect to FruBM function, given the pleiotropic nature of TRF-2. The neurite phenotypes could be due to the activities of other transcription factors that interact with TRF-2 in this population of neurons, including, for example, downstream target genes of FruBM that are transcription factors.
A6. In the revised manuscript, we present evidence that TRF2 directly binds to the promoter region of the *robo1* gene, an established direct transcriptional target of FruBM

(line 211-214, page 14; Fig. 5b). In addition, we have provided in vivo data that demonstrate that *Trf2*, *fru* and *robo1* genetically interact in the sex-type specification of mAL neurons (line 255-260, page 17; Supplementary Fig. 4). For example, we show that *Trf2* knockdown in fru^{GAL4}-expressing neurons phenocopied *robo1*[Δ1]/*robo1*[Δ3] mutants, by inducing the precocious wing-switching in courting males. The *robo1*[Δ1]/*robo1*[Δ3] mutants have deletions of a few nucleotides in the FruBM-binding site in the endogenous *robo1* gene, and thus the precocious wing-switching in these flies is considered to be specifically associated with the FruBM-mediated transcriptional regulation of *robo1* (see also A11-2).

Through these experiments, we have firmly demonstrated that the observed effects of manipulating *Trf2* represent specific roles of *Trf2* in the neural sex differentiation.

Q7. Their approach to show specificity for courtship is also not convincing, as they perform the knockdown of TRF-2 only in Fru neurons. Are Fru neurons required for locomotor behaviors?
A7. Locomotor activities in *Drosophila* are sexually dimorphic (e.g., Gatti et al., 2000, Curr. Biol. 10, 667-670) and some aspects of locomotor sexual differences have been linked to *fru* gene functions (e.g., Devineni and Heberlein, 2012, Proc. Natl. Acad. Sci. USA 109, 21087-21092).

Q8. In the tissue culture assay, the authors find that adding TRF-2 increased expression of a reporter through a 1.7KB upstream region of robo. Addition of FruBM reduced expression. In both these cases the differences were modest, though significant. The reduction by FruBM required a large amount of DNA to be transfected, and thus FruBM may be acting to sequester factors needed for transcription in a non-specific manner. In panel 4C there is no significant difference from mock, when FruBM or FruBM and TRF-2 are transfected. The modest significant differences are present only in comparison to TRF-2 transfection conditions, where there was a significant increase in reporter activity. There are many possible interpretations of this result.

Strong data supporting direct binding interactions on this DNA element are thus critical, which the authors attempt by gel shift assays. The gel shift assay does not have sharp bands, but is very smeary. The supershift in lane 5 after addition of FruBM is not very convincing. How repeatable is this result? Also, the authors do not have a lane with FruBM and not TRF-2. Interference by anti-TRF-2 antibody is not convincing either, given that addition of an antibody can interfere in non-specific ways. The proteins they made in the extracts were tagged. Did they try antibodies against the epitope tags? A supershift would be much more convincing.
A9. We carried out additional gel shift assays in which we used an anti-Tag antibody in place of the anti-TRF2 antibody for interference, confirming that TRF2 produces a retarded band that represents the complex of TRF2 and a *robo1* promoter fragment. The bands obtained are now all sharp and unambiguous. We have also added data indicating that an unrelated antibody (mouse IgG) does not interfere with formation of the TRF2-*robo1* complex, and that transfection with FruBM alone (without TRF2) does not produce a retarded band, provided that fragment-A is used as the DNA probe.

Q10.General comments:
Some of the language in the paper was redundant, especially topics in the introduction and beginning of results.
Lines 158-160 are very confusing.
Did the authors only look at 4 females brains? Line 170. That is a small N for this type of experiment.
A10. We have rewritten the passages to clarify the context. We have also increased the sample number.

Reviewer #3 (Remarks to the Author):

A major goal in behavioral neurogenetics is to establish causal links between the actions of developmental regulatory genes, the development of neurons and circuits, and the generation of innate behaviors. In Drosophila, male-specific products of the fruitless gene (fruM) are critical in building the neural potential for male courtship behavior. Fru encodes a BTB-containing transcription factor. How exactly fruM specifies male behavior has only recently started to become unraveled. The Yamamoto group has been a pioneer in this regard. Focusing on the sex-specific development of a defined fruM-expressing neural class, the mAL neurons, the Yamamoto group recently identified the robo1 gene as a direct transcriptional target of fruM (specifically the B isoform of fruM: fruBM). They demonstrated that fruBM represses the expression of robo1 in the mAL neurons, thereby allowing the development of a male-specific ipsilateral neurite. In the absence of fruBM, as in females, the mAL neurons fail to develop the male-specific neurite due to the expression of robo1.

The manuscript by Chowdhury et al is a substantial step forward in our understanding of the molecular mechanisms by which fruBM regulates robo1 expression. In this study, Chowdhury et al submit that fruBM regulates robo1 through the core promoter factor, Trf2. A combination of behavioral, neuroanatomical and biochemical experiments suggest that Trf2 promotes both male and female development based on the presence or absence of fruBM. More specifically, the authors propose an intriguing model wherein Trf2 promotes robo1 expression in females, whereas in males, the presence of fruBM ‘switches’ Trf2 into repressor, thereby shutting down robo1 expression. They propose that these interactions occur in cis at the robo1.

The work described in Chowdhury et al is well worth publication, as their conclusions are a big leap in our understanding of the molecular mechanisms by which fruM specifies sex-specific development. That said, the authors do not provide enough experimental support for their claims, and I highly recommend that they include additional experiments before publication. In particular, the manuscript lacks any in vivo genetic support for the interactions between fruBM, Trf2 and robo1. My concerns are enumerated below.

Q11. The authors claim that Trf2 activates robo1 expression in females, whereas in males, the presence of FruBM converts Trf2 into a repressor, thereby turning robo1

expression off. These molecular interactions account (at least in part) for the sex-specific development of the ipsilateral mAL neurite.

As the paper stands, the authors provide no in vivo genetic support for the link between FruBM, Trf2 and robo1. I recommend that the authors do include genetic experiments that test the proposed interactions between FruBM, Trf2 and robo1. For example:

Q11-1.(a) If Trf2 and FruBM repress robo1 expression in males, then the loss of the male-specific ipsilateral mAL neurite in Trf2 knockdown males should be dependent upon the gain of robo1 expression. The authors could test this by asking if the knockdown of robo1 rescues Trf2 mutant neuroanatomy in males.
A11-2. Thank you very much for the thoughtful suggestion. We have conducted an additional experiment to simultaneously knockdown *Trf2* and *robo1* in mAL single cell MARCM clones and test whether the loss of the male-specific ipsilateral neurite by *Trf2* knockdown is rescued by inhibiting *robo1*. As shown in Supplementary Fig. 4 of the revised manuscript, *robo1* knockdown seemed to impede the loss of the ipsilateral neurite induced by *Trf2* knockdown, although this effect was not large enough to be statistically significant. We have described and discussed this result in line 255-260, page 17.

Q11-2. (b) Based on their biochemical data, the authors claim that FruBM represses robo1 through Trf2. The idea is that FruBM ‘switches’ Trf2 from being an activator to being a repressor. If this were true, then the authors should be able to rescue the loss of the male-specific ipsilateral mAL neurite in FruBM mutants by knocking down Trf2 function. For instance, the Yamamoto group published a robo1 mutant that lacks the putative FruBM binding site. This mutant produces “dwarf” male-specific neurites. Is this suppressed by the concomitant loss of Trf2?
A11-2. Although the FruBM-binding site (FROS) is located close to the DREAM motif that we identified as the site where TRF2 interacts with the *robo1* promoter, it is possible that FruBM may exert its repressor effect without binding to the FROS, when FruBM is recruited to the *robo1* promoter via TRF2. Because of such potential complications, we decided to leave this problem for our future investigation.

Q11-3. (c) The authors may also be able to over-express fruBM in the mAL neurons of females, which would presumably cause a gain of the male-specific neurite. They can then ask whether the resulting phenotype is dependent upon the function of Trf2.
A11-3. In our experience, transgenic overexpression of FruBM via fru^{GAL4} drivers kills flies. For this reason, we were unable to carry out the suggested experiment.

The authors use luciferase reporter assays to provide in vitro evidence that Trf2 is an activator by itself, but a repressor in the presence of FruBM. As it stands now, this claim is established with just a single experiment. I recommend that the authors do additional experiments with mutant constructs to validate their results. For example:

Q12-1. (a) Luciferase reporter expression is repressed when Trf2 is co-transfected with low amounts of FruBM. Based on this result, the authors suggest that Trf2 becomes a repressor in the presence of fruBM. This is a substantial claim, and it deserves to be

buttressed with additional experiments. For instance, the authors could repeat this experiment using a mutant construct in which the putative fruBM binding site has been mutated. If what they are claiming is true, then reporter expression should increase above the baseline, as Trf2 would activate transcription.
A12-1. As pointed out in A11-3 above, the mechanism of TRF2 and FruBM interactions could be very complex, and we cannot exclude the possibility that FruBM might act on *robo1* transcription in a FROS-independent manner, provided that TRF2 is able to bind to the *robo1* promoter. We consider that this issue is beyond the scope of the current paper, which focuses primarily on the phenotypic outcome of TRF2-FruBM interactions on the *robo1* promoter.

Q12-2. (b) The repression observed when co-transfecting FruBM and Trf2 should be dependent upon the putative Trf2 binding site. This is testable: The authors could repeat the experiment in Fig 4C with the Trf2 binding site mutated; this should cause a loss of repression, restoring reporter expression to baseline levels.
A12-2. We now show that the DREAM motif in the *robo1* promoter is essential for the TRF2 action to enhance FruBM-mediated repression, whereas both TCT and DREAM motifs are required for the increase in the reporter activity. Thus these two functions of TRF2 are separable in terms of their molecular basis. These results are now mentioned in line 224-235, page 15 and Fig. 5d.

Q13. Wing switching. The authors should provide genetic evidence for the interactions between fruBM, Trf2 and robo1 in behavior. Previously, the Yamamoto group (Ito et al., 2016) was able to rescue the wing switching phenotype of robo1-delta1 mutants (these mutants carry mutations in the putative fruBM binding site, as noted above) by expressing an RNAi against robo1 (using 43D01-Gal4). This supported the notion that the delta1 mutation caused an upregulation of robo1 expression. Based on the data presented in the current manuscript, one might predict that the knockdown of Trf2 would also rescue the wing switching phenotype of robo1-delta mutants (b/c the gain of robo1 expression should be dependent upon Trf2.): 43D01-Gal4 -> UAS-Trf2-RNAi, robo1-delta1/+ versus robo1-delta1. Notably, if this experiment failed to rescue, it may add to the authors' idea that fruBM regulates target gene expression (including robo1) in a variety of ways. Also, the authors should tell us to what extent the loss of Trf2 in males phenocopies robo1-delta1 mutants.
A13. We found that *Trf2* knockdown phenocopies *robo1*[Δ1] mutants. The revised manuscript includes this new result. We added the following passage to describe this finding: "We also examined whether *Trf2* knockdown results in the precocious wing-switching, a change in the male courtship posture uniquely induced in male mutant flies (e.g., *robo1*[Δ1]/*robo1*[Δ3]) whose *robo1* gene lacks a few nucleotides that compose the core binding motif for FruBM binding. Strikingly, *Trf2* knockdown in *fru*^{GAL4}-expressing neurons phenocopied the *robo1*[Δ1]/*robo1*[Δ3] mutants by exhibiting the precocious wing-switching (Supplementary Fig. 5). These results are consistent with the idea that the disturbance of sex-specific traits by *Trf2* knockdown is, at least in part, expressed through the mis-expression of *robo1* in *fru*-positive neurons including mAL neurons".
In our additional experiments, we further demonstrated that *robo1* knockdown significantly reduced the precocious wing-switching induced by *Trf2* knockdown

(Supplementary Fig. 5 and line 264–267, page 17 and line 268-270, page 18). Please also see A11-2 for *robo1* knockdown effects on mAL sex-type changes induced by *Trf2*-knockdown.

Q14. 4. Does Trf2 contribute to female behavior? The authors provide evidence that Trf2 promotes male behavior and neuroanatomy in the presence of FruM. They also provide evidence that Trf2 feminizes neuroanatomy, but leave female behavior out. The authors should attempt to knockdown Trf2 in females using fruGal4, for example, and asking whether or not female receptivity or egg laying is affected.
A14. Our additional experiment showed that *Trf2* knockdown via fru^{GAL4} significantly reduced female receptivity and egg laying. This result is described in line 129-132, page. 9; and Supplementary Fig. 2 in the revised manuscript (please see also A3).

Reviewers' comments:

Reviewer #1 (Remarks to the Author):

I have no further questions to Authors and would like to recommned the MS for publication.

Reviewer #2 (Remarks to the Author):

Response to the authors (reviewer 2):

1. The authors response about the "validity of the genetic screen" did not address the concern raised. It is clear that they have identified a suppressor of the rough eye phenotype caused by over-expression of FruB in a valid genetic screen. However, the authors make strong claims about the "specificity of the interaction", which is their argument for unique interactions between Trf-2 and FruB.

I don't think the authors can say anything about the specificity (uniqueness) of the Trf-2/FruB interaction, by showing they do not observe a similar phenotypic suppression of a Tramtrack induced rough eye phenotype. They only tested one other transcription factor (Tramtrack) and even for this one transcription factor there are trivial experimental reasons why they may not see suppression, even if Trf-2 and Tramtrack interact (for example, it may be more highly expressed than FruB). A single negative result is not sufficient to firmly say that Trf-2 specifically interacts with Fru, as they do on lines 117-119.

With respect to this genetic screen, it will identify what they screened for---genes that reduce the rough eye phenotypes caused by overexpression of a transcription factor. Thus, a gene that encodes a protein that interacts with core transcriptional machinery is likely to be discovered in this screen, was the point I was making. Furthermore, they know from other studies that Trf-2 is likely a broadly used co-transcription factor.

The result of this screen is still very interesting, but they should describe it more accurately. They have an interesting finding, especially their observation that Trf-2 can be a co-activator or a co-repressor, in a context-specific manner. I think emphasizing the specificity of the interaction with FruBM is incorrect, as they have not shown that. Perhaps they can use language that emphasizes that they understand Trf-2 mechanistic functions with more specificity?

2. Again, I don't think the authors address the concern raised in their response, even though the authors provide results pointing to a role of FruBM/Trf-2/robo being involved in male behavior and mAL neuronal morphology.

The behavior and neuronal morphological data are based on RNAi reduction of Trf-2 in fru neurons using Fru-Gal4. Thus, the authors do not know it is through Trf-2/FruBM interactions in those experiments and so they should not use language to suggest their data shows this result. This is an issue in the discussion and the results.

To be clear, the phenotype could be a result of Trf-2 working with other transcription factors, even other fru isoforms. Their genetic interaction/"interrelationships" experiments don't show that all fru or Trf-2 mAL and behavioral phenotypes are through robo, as they note in the results, so they should not assume that what they observe in vitro by gel shift and in the tissue culture experiments can account for all the in vivo observations.

3. The authors state that FruBM acts to repress transcription in males and activate transcription in females on target genes (lines 90-93). They need to make it clear that they mean robo and not all FruBM target genes. I found a reference that suggests FruBM is an activator, so that sentence is very confusing (Male-specific Fruitless isoforms have different regulatory roles conferred by distinct zinc finger DNA binding domains.). Furthermore, it is hard to move between the different papers, as this laboratory uses one nomenclature that is not used in some other papers. Can the authors provide both fru nomenclatures in the paper.

4. On line 101, they reference #31 as showing that FruBM is the most prevalent isoform in neural masculinization. I looked for this result in reference #31 and could not find it.

5. When the authors use the Fru-FLP transgene in combination with Trf-2 Gal4, why are only a subset of mAL Fru+ neurons labeled? There are red neurons in the mAL region, without the surrounding green label. They argue that all mAL neurons are Fru+/Trf-2+. In the antibody staining experiment, it is not clear that the mAL neurons all are Fru+/Trf-2+. Can they authors provide higher magnification?

6. The authors cite reference #27 for Fru being a repressor of ~140 target genes. I looked at this reference and did not see the relevant data.

7. I stil find lines 147-152 very confusing.

8. The authors use poxn-Gal4, but don't say how much it overlaps with fru expression. They did not provide a reference for this, so I could not look it up easily.

Overall, the authors have interesting in vivo and in vitro data on a Fru interacting protein and how it acts with Fru on a single target gene with different activities that depend on context. However, I find that the authors overly generalize their findings about the single target gene to explain the morphological and behavioral data that is likely a result of Trf-2 and Fru acting on many target genes. This seems like something the authors can fix by rewriting portions of the manuscript.

Reviewer #3 (Remarks to the Author):

The authors have addressed the concerns I described in my review to my satisfaction. I am happy to see it in press.

Point-by-point replies to the reviewer's comments

Reviewers' comments:

Reviewer #1 (Remarks to the Author):

I have no further questions to Authors and would like to recommned the MS for publication.

Reviewer #2 (Remarks to the Author):

Response to the authors (reviewer 2):

Q1. The authors response about the "validity of the genetic screen" did not address the concern raised. It is clear that they have identified a suppressor of the rough eye phenotype caused by over-expression of FruB in a valid genetic screen. However, the authors make strong claims about the "specificity of the interaction", which is their argument for unique interactions between Trf-2 and FruB.

I don't think the authors can say anything about the specificity (uniqueness) of the Trf-2/FruB interaction, by showing they do not observe a similar phenotypic suppression of a Tramtrack induced rough eye phenotype. They only tested one other transcription factor (Tramtrack) and even for this one transcription factor there are trivial experimental reasons why they may not see suppression, even if Trf-2 and Tramtrack interact (for example, it may be more highly expressed than FruB). A single negative result is not sufficient to firmly say that Trf-2 specifically interacts with Fru, as they do on lines 117-119.

With respect to this genetic screen, it will identify what they screened for---genes that reduce the rough eye phenotypes caused by overexpression of a transcription factor. Thus, a gene that encodes a protein that interacts with core transcriptional machinery is likely to be discovered in this screen, was the point I was making. Furthermore, they know from other studies that Trf-2 is likely a broadly used co-transcription factor.

The result of this screen is still very interesting, but they should describe it more accurately. They have an interesting finding, especially their observation that Trf-2 can be a co-activator or a co-repressor, in a context-specific manner. I think emphasizing the specificity of the interaction with FruBM is incorrect, as they have not shown that. Perhaps they can use language that emphasizes that they understand Trf-2 mechanistic functions with more specificity?

A1. We have deleted the sentence arguing about the specificity of interactions between fru and Trf-2 overexpressed in the eye disc (the deleted part corresponds to lines 117-119 of the R1 manuscript).

Q2. Again, I don't think the authors address the concern raised in their response, even though the authors provide results pointing to a role of FruBM/Trf-2/robo being involved in male behavior and mAL neuronal morphology.

The behavior and neuronal morphological data are based on RNAi reduction of Trf-2 in fru neurons using Fru-Gal4. Thus, the authors do not know it is through Trf-2/FruBM interactions in those experiments and so they should not use language to suggest their data shows this result. This is an issue in the discussion and the results.

To be clear, the phenotype could be a result of Trf-2 working with other transcription factors, even other fru isoforms. Their genetic interaction/"interrelationships" experiments don't show that all fru or Trf-2 mAL and behavioral phenotypes are through robo, as they note in the results, so they should not assume that what they observe in vitro by gel shift and in the tissue culture experiments can account for all the in vivo observations.

A2. We have toned down our statement by adding the passage “It remains to be determined whether TRF2 regulates transcription of the FruM-target genes other than robo1 and, if so, what aspects of sex-specific neurobehavioral traits are controlled by such mechanisms.” (lines 265 – 268, Pages. 17-18)

Q3-1. The authors state that FruBM acts to repress transcription in males and activate transcription in females on target genes (lines 90-93). They need to make it clear that they mean robo and not all FruBM target genes.

A3-1. We have rephrased the relevant passage to clarify that we are dealing with a specific target gene (i.e., robo1) and not FruBM target genes in general. The rephrased passage is : “We propose that TRF2 governs sex-specific function of a FruBM target gene, *robo1*, from that of an activator to a repressor upon detection of the presence of FruBM in males.” (lines 90 – 92, Pages 6-7).

Q3-2. I found a reference that suggests FruBM is an activator, so that sentence is very confusing (Male-specific Fruitless isoforms have different regulatory roles conferred by distinct zinc finger DNA binding domains.).

A3-2. Some studies infer that FruM isoforms play an activator role in transcription, but direct evidence for this is lacking.

Q3-3. Furthermore, it is hard to move between the different papers, as this laboratory uses one nomenclature that is not used in some other papers. Can the authors provide both fru nomenclatures in the paper.

A3-3. We have included the description on an alternative nomenclature used by other groups to refer to the FruM isoforms in the following sentence: “Thus the P1 promoter-derived transcripts encode male-specific Fru isoforms, FruAM, FruBM, FruCM, FruDM and FruEM (M stands for male-specific and A-E indicate the C-terminal variant types; Isoforms A, B and E in our nomenclature correspond to isoforms A, C and B in the nomenclature adopted by von Philipsborn et al.), among which FruBM (FruCM by von Philipsborn et al.) is the isoform with the strongest impact on neurobehavioral masculinization.” (lines 54 – 59, Page 4.)

Q4. On line 101, they reference #31 as showing that FruBM is the most prevalent isoform

in neural masculinization. I looked for this result in reference #31 and could not find it.

A4. We have rephrased as “Fru BM is the isoform with the strongest impact on neurobehavioral masculinization”, based on von Phillipsborn et al. (2014, Curr. Biol. 24, 242-251). (lines 58 – 59, Page. 4).

Q5. When the authors use the Fru-FLP transgene in combination with Trf-2 Gal4, why are only a subset of mAL Fru+ neurons labeled? There are red neurons in the mAL region, without the surrounding green label. They argue that all mAL neurons are Fru+/Trf-2+. In the antibody staining experiment, it is not clear that the mAL neurons all are Fru+/Trf-2+. Can they authors provide higher magnification?

A5. We infer that the flippase was effective in not all the cells where fru[FLP] was expressed, resulting in a few marker-negative cells in the mAL cluster. In the revised manuscript, we have included an enlarged image of the mAL cluster doubly stained for FruM and TRF2 (Supplementary Figure 3 c-e).

Q6. The authors cite reference #27 for Fru being a repressor of ~140 target genes. I looked at this reference and did not see the relevant data.

A6. We have corrected the reference number as 25. We apologize for this error.

Q7. I stil find lines 147-152 very confusing.

A7. We have rephrased as “When the mAL cluster was visualized as a neuroblast clone, the male-specific ipsilateral neurite appeared shorter in males with *Trf2* knockdown than in control males (Fig. 2a, b). mAL neuroblast clones in *fru* hypomorphic mutant males exhibited a similar shortening of the ipsilateral neurite, which was ascribed to a reduction in the number of mAL neurons with a long ipsilateral neurite as resulting from sexual transformation of single cells.” (lines 141- 147, Page. 10).

Q8. The authors use poxn-Gal4, but don't say how much it overlaps with fru expression. They did not provide a reference for this, so I could not look it up easily.

A8. Mellert et al. (2010, Development 137, 323-332) reported that 96% of poxn-GAL4-positive sensory cells in forelegs are fru-positive. We have described this fact with the citation of Mellert et al. in lines 487 – 488, Page. 31 of the revised manuscript.

Q9. Overall, the authors have interesting in vivo and in vitro data on a Fru interacting protein and how it acts with Fru on a single target gene with different activities that depend on context. However, I find that the authors overly generalize their findings about the single target gene to explain the morphological and behavioral data that is likely a result of Trf-2 and Fru acting on many target genes. This seems like something the authors can fix by rewriting portions of the manuscript.

A9. We hope that overstated phrases in the previous manuscript have been adequately fixed by the above revisions.

Reviewer #3 (Remarks to the Author):

The authors have addressed the concerns I described in my review to my satisfaction. I am happy to see it in press.

REVIEWERS' COMMENTS:

Reviewer #2 (Remarks to the Author):

I appreciate the authors' responses.

However, I looked through the paper they cite in one respone and can not find strong evidence for the following statement pasted below from the response letter.

First, I am not sure how they came up with 140 genes from the salivary gland analyses and then infer that is true in the CNS. Second, there is no expression data in that paper, so it is not clear how they can say if Fru has repressor or activator functions. Close proximity to a histone mark/modifier based on salivary gland squash data is not sufficient to make that claim. Also, those experiments do not have good resolution. Finally, it looks like they are examining a different FRU isoform in that paper, as the letter designation is different. If there are other sentences in the paper that make this claim those should be removed too.

Otherwise, the authors' responded to the points raised.

FROM RESPONSE LETTER:
Q6. The authors cite reference #27 for Fru being a repressor of ~140 target genes. I looked at this reference and did not see the relevant data.

A6. We have corrected the reference number as 25. We apologize for this error.

Point-by-point replies to the reviewer's comments

The reviewer's comments
Reviewer #2 (Remarks to the Author):

I appreciate the authors' responses.

However, I looked through the paper they cite in one respone and can not find strong evidence for the following statement pasted below from the response letter.

First, I am not sure how they came up with 140 genes from the salivary gland analyses and then infer that is true in the CNS. Second, there is no expression data in that paper, so it is not clear how they can say if Fru has repressor or activator functions. Close proximity to a histone mark/modifier based on salivary gland squash data is not sufficient to make that claim. Also, those experiments do not have good resolution. Finally, it looks like they are examining a different FRU isoform in that paper, as the letter designation is different. If there are other sentences in the paper that make this claim those should be removed too.

Otherwise, the authors' responded to the points raised.

FROM RESPONSE LETTER:
Q6. The authors cite reference #27 for Fru being a repressor of ~140 target genes. I looked at this reference and did not see the relevant data.

A6. We have corrected the reference number as 25. We apologize for this error.

Our replies
We have removed the relevant passages in the main text, i.e., lines 19 - 20, P. 2 and lines 304 – 313, P. 20 of our R2 manuscript.

In addition, we have removed the sentence "We found no obvious shortening of the ipsilateral neurite in single cell clones of mAL upon *Trf2* knockdown (data not shown)" (lines 157 - 158, P. 11 of our R2 manuscript) as well as the passage "We first tested a

robo1 promoter fragment of 120 bp (the B fragment) that contains the FruBM-binding motif (FROS) (Fig. 5a), and found no shifted band. In contrast, …(data not shown)” (lines 206 - 208, P. 14 of our R2 manuscript), according to the editor’s suggestion.
